# A Novel OsMPK6‐OsMADS47‐PPKL1/3 Module Controls Grain Shape and Yield in Rice

**DOI:** 10.1002/advs.202501946

**Published:** 2025-06-05

**Authors:** Jingjing Fang, Yan Chun, Fan Zhang, Tingting Guo, Mengmeng Ren, Jinfeng Zhao, Shoujiang Yuan, Wensheng Wang, Yunhai Li, Xueyong Li

**Affiliations:** ^1^ State Key Laboratory of Crop Gene Resources and Breeding National Key Facility for Crop Gene Resources and Genetic Improvement Institute of Crop Sciences Chinese Academy of Agricultural Sciences Beijing 100081 China; ^2^ College of Agriculture and Biotechnology Hunan University of Humanities Science and Technology Loudi 417000 China; ^3^ Institute of Wetland Agriculture and Ecology Shandong Academy of Agricultural Sciences Jinan 250100 China; ^4^ State Key Laboratory of Plant Cell and Chromosome Engineering CAS Centre for Excellence in Molecular Plant Biology Institute of Genetics and Developmental Biology The Innovative Academy of Seed Design Chinese Academy of Sciences Beijing 100101 China

**Keywords:** grain shape, kelch‐repeat protein phosphatase, MADS‐box transcription factors (TFs), MAPK kinase, rice

## Abstract

Grain shape is a key determinant of both grain yield and appearance quality in rice. However, the interactions among different regulatory pathways remain unclear. Here, a novel regulatory axis centered on a MADS‐box transcription factor (TF) OsMADS47 for controlling rice grain shape is reported. *OsMADS47* overexpression results in slender grains, whereas knockout leads to short and small grains. OsMADS47 acts downstream of the OsMKKK10‐OsMKK4‐OsMPK6 cascade and can be phosphorylated by OsMPK6. Phosphorylation stabilizes OsMADS47 and enhances its transcriptional repression activities on target genes *GS3* and *GW8*, two well‐known grain shape regulators. Meanwhile, the Kelch‐repeat protein phosphatase PPKL1/3 can dephosphorylate OsMADS47, making it unstable and releasing the transcriptional repression on target genes. Knockdown of *OsMPK6* or overexpression of *PPKL1/3, GS3*, or *GW8* partially suppresses the slender‐grain phenotype of the *Os* *MADS47* overexpression plants. The results establish an integrated regulation pathway of grain shape and prove the hub gene *OsMADS47* as a potential target for breeding rice with optimized appearance quality.

## Graphical abstract

OsMADS47 is identified as a key regulator governing rice grain morphology. Phosphorylation‐dependent regulation of its activity directs the development of slender versus round grains, modulating both yield and appearance. This mechanism bridges cellular signaling with transcriptional regulation, establishing a molecular framework for grain optimization. The findings offer an actionable genetic target for developing high‐yielding rice varieties with improved grain appearance.

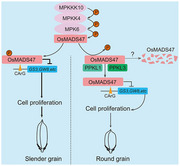

## 1 Introduction

Grain shape is an important determinant of grain yield and appearance quality.^[^ ^1^ ^]^ Slender grains are highly commercial worldwide and preferred by the majority of consumers, especially in Southeast Asia.^[^ ^2^ ^]^ However, slender grains are usually associated with some negative effects on grain yield, such as decreased grain number per panicle.^[^ ^1^ ^]^ Thus, it is a challenge to improve grain yield and appearance quality simultaneously.^[^ ^3^ ^]^

In recent years, many quantitative trait loci (QTLs) and genes for grain size and/or shape have been cloned from different rice germplasms and molecularly characterized. E.g., *GS3*, *qGL3*/*GL3.1*/*qGL3‐1*, *TGW6*, *OsLG3*, *GLW7*, *GL4*, *qLGY3*/*OsLG3b*/*GW3p6*, and *TGW3*/*qTGW3*/*GL3.3* are the major QTLs controlling grain length.^[^ ^2^, ^3^, ^4^, ^5^, ^6^, ^7^, ^8^ ^]^ *GW2*, *qSW5*/*GW5*/*GSE5*, *GS5*, *GW8*, and *TGW2* were identified as main regulators of grain width.^[^ ^9^, ^10^, ^11^, ^12^, ^13^ ^]^ Additionally, several QTLs controlling both grain length and width have been isolated, such as *GL2/GS2*, *GL7/GW7/SLG7*, *GW6a*, and *GS9*.^[^ ^9^, ^14^, ^15^, ^16^ ^]^ Functional analysis of these major QTLs and a number of other genes for grain size and/or shape sheds light on the molecular mechanisms of grain size regulation.

It was reported that the identified genes regulate grain shape mainly through several specific processes, such as G‐protein‐coupled signaling pathway, ubiquitination, transcription regulators, MAPK signaling pathway, and phytohormones.^[^ ^17^ ^]^ G‐protein regulation pathway transmits signal to downstream effectors via a membrane receptor (GPCRs) and the heterotrimeric G‐protein complex consisting of Gα, Gβ, and Gγ subunits.^[^ ^18^ ^]^ There are one Gα (*RGA1*), one Gβ (*RGB1*), and five Gγ (*RGG1*, *RGG2*, *GS3*, *DEP1*, and *GGC2*) genes in the rice genome.^[^ ^18^ ^]^ The Gγ subunits GS3 and DEP1 interact directly with OsMADS1 and cooperatively enhance its transcriptional activity on common target genes, thereby regulating grain size and shape.^[^ ^3^ ^]^ Additionally, the HGW, GW2, WTG1, and OsUBP15 proteins regulate grain size and weight through the ubiquitin‐proteasome pathway.^[^ ^9^, ^19^, ^20^, ^21^ ^]^ Transcriptional factors also play important roles in controlling grain size, such as GW8/SPL16, GLW7, and GS9.^[^ ^1^, ^14^, ^15^ ^]^ *GW8*/*SPL16* encoding a SQUAMOSA PROMOTER BINDING PROTEIN‐LIKE (SPL) transcription factor (TF) is a major QTL which positively regulates grain width and yield via influencing cell proliferation in the grain‐width direction.^[^ ^1^ ^]^ GW8/SPL16 binds directly to the promoter of *GL7* and represses its expression.^[^ ^12^ ^]^

Recent studies have highlighted the importance of MAPK (mitogen‐activated protein kinase) signaling pathway in the control of grain shape. A MAPK cascade usually consists of at least three types of activated protein kinases: an MAPK kinase (MAPKKK, MKKK, or MEKK), an MAPK kinase (MAPKK, MKK, or MEK), and an MAPK.^[^ ^22^ ^]^ The MAPK cascades play key roles in transducing exogenous or developmental signals to downstream effectors by sequential phosphorylation to regulate multiple processes, including defense responses, plant growth, and development.^[^ ^23^, ^24^, ^25^ ^]^ The OsMKKK10‐OsMKK4‐OsMPK6 cascade positively regulates grain size and weight.^[^ ^26^ ^]^ Overexpression of a constitutively active *OsMPKKK10* resulted in large and heavy grains, while loss of *OsMPKKK10* function generated opposite grain morphology.^[^ ^26^ ^]^ The OsMKKK10‐OsMKK4‐OsMPK6 cascade was negatively regulated by the mitogen‐activated protein kinase phosphatase GSN1 (GRAIN SIZE AND NUMBER1) which coordinates the trade‐off between grain number and grain size by directly dephosphorylating OsMPK6.^[^ ^27^ ^]^ *OsRac1* encodes a ROP GTPase which phosphorylates OsMPK6 to positively regulate grain size by regulating cell division.^[^ ^28^ ^]^ Overexpression of *OsRac1* not only results in a larger spikelet hull, but also causes greater grain width and weight by accelerating grain‐filling rate.^[^ ^28^ ^]^ Further studies identified components upstream and downstream of the GSN1‐MAPK (GSN1‐OsMKKK10‐OsMKK4‐OsMPK6) module. ERECTA1 (OsER1), a receptor‐like protein kinase, acts upstream of the OsMKKK10‐OsMKK4‐OsMPK6 cascade to regulate spikelet number per panicle by maintaining cytokinin homeostasis.^[^ ^29^ ^]^ Downstream of this MAPK cascade, OsMPK6 directly phosphorylates DST, a zinc finger TF, and enhances its transcriptional activation activity on *CYTOKININ OXIDASE2* (*OsCKX2*) to modulate cytokinin metabolism.^[^ ^30^ ^]^ Plant hormones such as brassinosteroids (BRs), auxins and cytokinins also directly or indirectly control grain size and shape. E.g., *GL3*/*GL3.1*/*OsPPKL1* encodes a Ser/Thr phosphatase with Kelch‐like domains (PPKLs) which might be involved in BR signaling.^[^ ^31^ ^]^ GL3/GL3.1/OsPPKL1 negatively regulates grain length by dephosphorylating cyclin‐T1;3 (CycT1;3) to affect the cell cycle progression.^[^ ^4^ ^]^ An aspartate‐to‐glutamate transition in the conserved AVLDT motif of the second Kelch domain in OsPPKL1 leads to long‐grain phenotype.^[^ ^32^ ^]^ GL3/OsPPKL1 suppresses the BR signaling by dephosphorylating and stabilizing OsGSK3 in rice.^[^ ^33^ ^]^

Although a large number of genes regulating grain size have been identified and functionally characterized, the interactions between different regulation pathways of grain shape remain largely unknown. It is essential to clone and characterize additional QTLs/genes involved in grain shape regulation. Grain size and number are inherently associated with floral organ identity which is mainly determined by various combinations of MADS‐box TFs.^[^ ^34^ ^]^ Plant MADS‐box TFs are a huge family and can be classified into M‐type and MICK‐type.^[^ ^35^ ^]^ To date, detailed analysis of MADS‐box proteins has been limited to the MIKC‐type. In addition to *OsMADS1* mentioned above, *OsMADS17*, *OsMADS51*, *OsMADS56*, and *OsMADS87* were identified to affect grain shape and yield, while the underlying molecular mechanisms remain unknown.^[^ ^3^, ^35^, ^36^, ^37^, ^38^, ^39^ ^]^

Here, we identified and characterized a new MADS‐box family member OsMADS47 involved in grain shape regulation. Overexpression of *OsMADS47* results in slender grains by affecting cell division patterns in the rice spikelet hull, and moderate expression of *OsMADS47* can also improve grain yield per plant. Further studies showed that OsMADS47 directly binds to the promoters of *GS3* and *GW*8, and represses their expression to regulate grain shape. OsMADS47 interacts with and can be phosphorylated by OsMPK6. The phosphorylated OsMADS47 becomes stable and enhances its transcription repression activities on *GS*3 and *GW8*. PPKL1/3 can dephosphorylate OsMADS47, respectively, to balance its phosphorylation level in rice. This fine‐tuned phosphorylation status of OsMADS47 by OsMPK6 and PPKL1/3 can effectively control grain shape through modulating the expression level of *GS3* and *GW8*. Thus, our findings reveal a novel molecular framework to control grain shape. More importantly, we provide a potential strategy for simultaneously improving both grain yield and appearance quality by designing *OsMADS47* directionally.

## 2 Results

### 2.1 OsMADS47 Regulates Grain Shape in Rice

**Figure 1:**
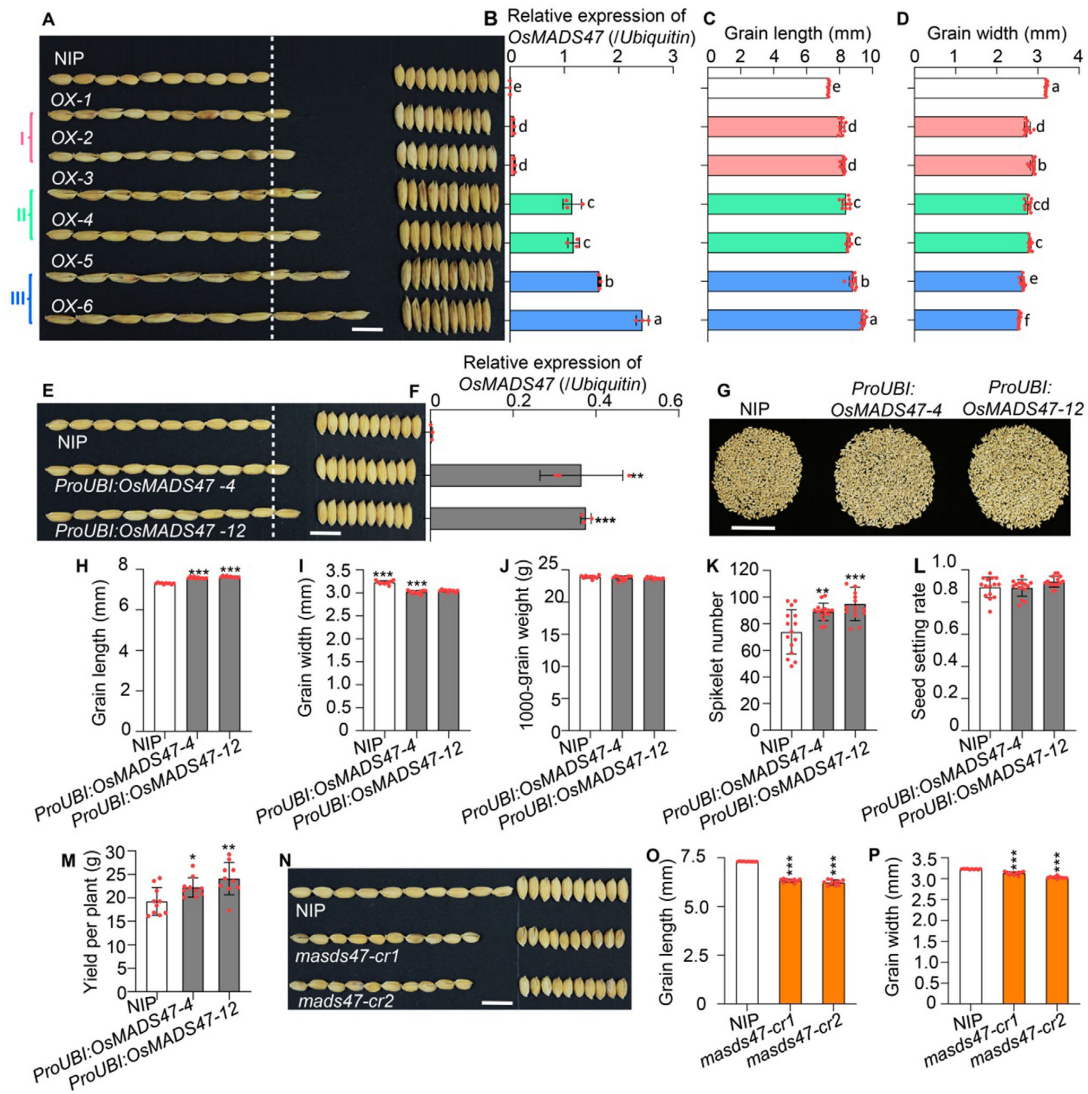
*OsMADS47* regulates grain length and width. A) Grain morphology of Nipponbare (NIP) and *OsMADS47* overexpression lines. Transgenic lines are designated as class I (*OX‐1* and *OX‐2*), II (*OX‐3* and *OX‐4*), and III (*OX‐5* and *OX‐6*) according to the levels of phenotypic change. The white dashed line indicates the total grain length of 10 grains in NIP. Scale bar, 1 cm. B) Relative expression of *OsMADS47* in NIP and *OsMADS47*‐overexpression lines, normalized to the rice *Ubiquitin* gene (*n* = 3). C) Grain length and D) grain width of NIP and *OsMADS47*‐overexpression lines (*n* = 10). In (B–D), data are given as mean ± SD. Different lowercase letters above bars indicate significant differences (*p* < 0.05) based on a one‐way ANOVA test. E) Grain morphology of NIP and *OsMADS47* overexpression plants driven by the maize *Ubiquitin1* promoter (*ProUBI:OsMADS47‐4* and *ProUBI:OsMADS47‐12*). The white dashed line indicates the total grain length of 10 grains in NIP. Scale bar, 1 cm. F) Relative expression of *OsMADS47* in NIP, *ProUBI:OsMADS47‐4* and *ProUBI:OsMADS47‐12*, normalized to the rice *Ubiquitin* gene (*n* = 3). G) Total grains per plant in NIP, *ProUBI:OsMADS47‐4* and *ProUBI:OsMADS47‐12*. Scale bar, 5 cm. H) Grain length, I) grain width, J) 1000‐grain weight, K) spikelet number, L) seed setting rate, and M) grain yield per plant in NIP, *ProUBI:OsMADS47‐4* and *ProUBI:OsMADS47‐12* (*n* = 10 in H–J and M; *n* = 15 in K and L). N) Grain morphology of *OsMADS47* knockout lines (*mads47‐cr1* and *mads47‐cr2*) generated by CRISPR/Cas9. Scale bar, 1 cm. O) Grain length and P) Grain width of *mads47‐cr1* and *mads47‐cr2* plants (*n* = 10). In F, H–M, O, and P, data are given as mean ± SD. Student's *t‐*test was used to generate the *p* values; *p < 0.05, **p < 0.01, ***p < 0.001.

To identify potential grain shape regulators from the MADS‐box protein family, we searched the Rice Genome Annotation Project database (RAP‐DB) and found a total of 78 MADS‐box genes in the rice genome. We selected 34 MADS‐box genes highly expressed in the developing panicles for overexpression driven by the rice *Actin1* promoter in a *japonica* rice variety Nipponbare (NIP) background. Besides *OsMADS1* and *OsMADS56*, which are known positive regulators of grain length,^[^ ^3^, ^35^, ^36^, ^37^, ^38^, ^39^ ^]^ overexpression of *OsMADS47* also resulted in long and slender grains (16 out of 21 lines). *OsMADS47* overexpression lines were grouped into three classes (I, II, and III) according to statistically significant differences in grain length (**Figure** **1**A,C). Class III exhibited the most pronounced grain length increase (≈24.5% vs the wild type NIP) with the highest intra‐class variation among transgenic lines (Figure 1A,C). Compared with NIP, the grain length of classes I and II increased by ≈11.7% and ≈15.5%, respectively, while their grain width decreased by ≈12.2% and ≈13.0% (Figure 1C,D). The extent of increase in grain length was positively correlated with the expression level of *OsMADS47* (Figure 1A–C). In addition, the *OsMADS47* overexpression plants showed reduced plant height and more tillers compared with NIP (Figure S1A,B,G, Supporting Information). Interestingly, although moderate overexpression of *OsMADS47* (*OX3* and *OX4*) had slight effects on thousand‐grain weight (TGW), the increased spikelet number per panicle without negative effects on seed setting rate led to a significant increase in grain yield per plant (Figure S1C–F,H, I, Supporting Information). To confirm this, we overexpressed *OsMADS47* in NIP driven by another strong and constitutive promoter, i.e., the maize *Ubiquitin1* promoter. The expression level of *OsMADS47* in transgenic lines (*ProUBI:OsMADS47‐4* and *ProUBI:OsMADS47‐12*) becomes significantly lower, compared with *OX‐5* and *OX‐6* with extremely high levels of *OsMADS47* (Figure S2, Supporting Information). Consistently, the transgenic plants showed moderate change in grain length and width, resulting in a higher grain yield per plant than NIP, suggesting that *OsMADS47* is a potential candidate gene to increase grain yield (Figure 1E–M).

To further verify the role of *OsMADS47* in regulating grain shape, we generated *OsMADS47* knockout plants (*mads47‐cr1* and *mads47‐cr2*) using CRISPR/Cas9 genome editing technology in the NIP background. *mads47‐cr1* harbored a 10‐bp deletion while *mads47‐cr2* had a 1‐bp insertion (Figure S3A, Supporting Information). Both knockout lines exhibited smaller grains with significant decreases in grain length and width, compared with NIP (Figure 1N–P). Additionally, *mads47‐cr1* and *mads47‐cr2* showed significant reductions in plant height, leaf length, panicle length, and spikelet number per panicle (Figure S3B–H, Supporting Information). Taken together, these results suggest that *OsMADS47* regulates both grain shape and plant morphology in rice.

### 2.2 *OsMADS47* Regulates Grain Shape by Altering Cell Division in Spikelet Hulls

**Figure 2:**
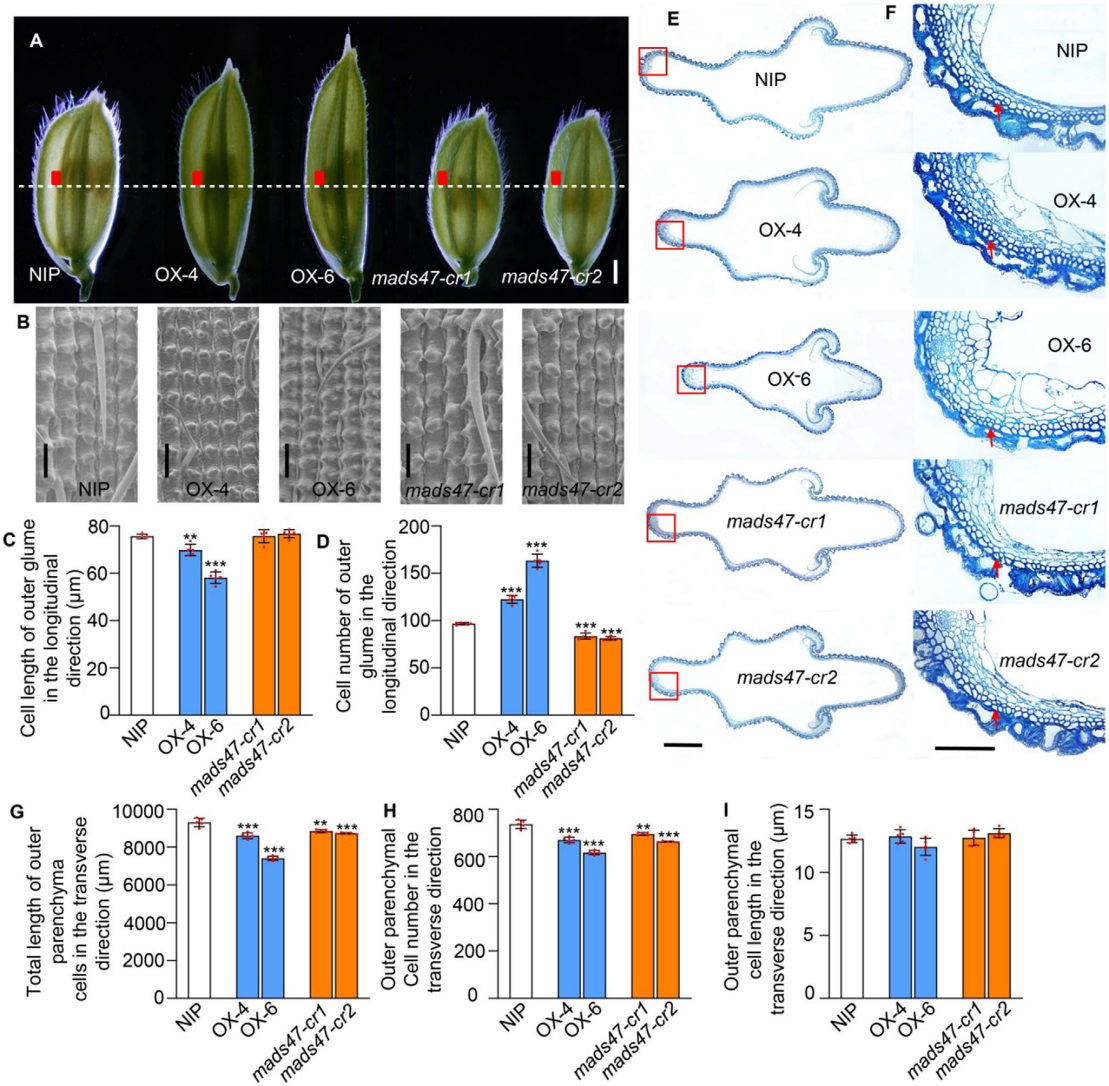
*OsMADS47* controls grain length and width by influencing cell proliferation. A) Spikelet hulls before anthesis in NIP and *OsMADS47* transgenic lines (*OX‐4*, *OX‐6*, *mads47‐cr1*, and *mads47‐cr2*). Scale bar, 1 mm. B) Scanning electron microscopy images of the outer surface of the red rectangular areas in (A). Scale bars, 100 µm. C) The average cell length and D) number of outer glume in the longitudinal direction in NIP and *OsMADS47* transgenic lines (*n* = 4). E) Cross‐sections of spikelet hulls indicated by the dashed line in (A). Scale bar, 100 µm. F) Magnified view of each boxed cross‐section shown in (E). Scale bar, 100 µm. G) Average total length, H) cell number, and I) cell length in the outer parenchymal cell layer (indicated by red arrow) in the transverse direction (*n* = 4). In C, D, and G–I, data are given as mean ± SD. Student's *t*‐test was used to generate the *p* values; **p < 0.01, ***p < 0.001.

Grain shape is molded by spikelet hull, which is regulated by cell proliferation and expansion. Thus, we first analyzed the outer glume cells in the longitudinal direction before fertilization by scanning electron microscopy. The average cell length of outer glumes in the *OsMADS47* overexpression lines (*OX‐4* and *OX‐6*) was significantly decreased, while no difference was found in *OsMADS47* knockout plants (*mads47‐cr1* and *mads47‐cr2*), compared with wild type (WT), NIP (**Figure** **2**A–C). Conversely, the cell number of outer glumes was significantly increased in *OsMADS47* overexpression lines while significantly decreased in *OsMADS47* knockout plants (Figure 2D). In addition, observations of cross‐sections showed that *OsMADS47* overexpression lines exhibited a significant decrease in the total length of outer parenchyma cells, while *mads47‐cr1* and *mads47‐cr2* also showed an obvious decrease possibly due to the essential role of *OsMADS47* in normal grain development (Figure 2E–G). The number of outer parenchyma cells was greatly decreased in *OsMADS47* overexpression plants while there was no difference in the average cell length compared with WT (Figure 2E,F,H,I). These observations implied that *OsMADS47* regulates grain length and width mainly through promoting cell division in the longitudinal direction while inhibiting cell division in the transverse direction.

Consistently, we found that the percentage of S and G2/M cells with higher DNA content was elevated in *OsMADS47* overexpression plants *OX‐4* and *OX‐6* (Figure S4A,B, Supporting Information). qRT‐PCR analysis of the cell‐cycle related genes including G1/S‐phase genes *MCM2*, *MCM3*, *MCM4*, *MCM5*, and *CDT2*, and G2/M‐phase genes *MAPK*, *CycB1;1* and *CDKB1;1*, were greatly increased in *OsMADS47* overexpression plants and obviously decreased in *OsMADS47* knockout plants, compared with NIP (Figure S4C, Supporting Information). These results demonstrated that *OsMADS47* might positively modulate the expression of some cell cycle genes to promote mitotic cell division during the early stages of spikelet development.

### 2.3 *OsMADS47* Highly Expresses in Young Tissues and OsMADS47 Localizes in the Nucleus to Function as a Transcriptional Repressor

**Figure 3:**
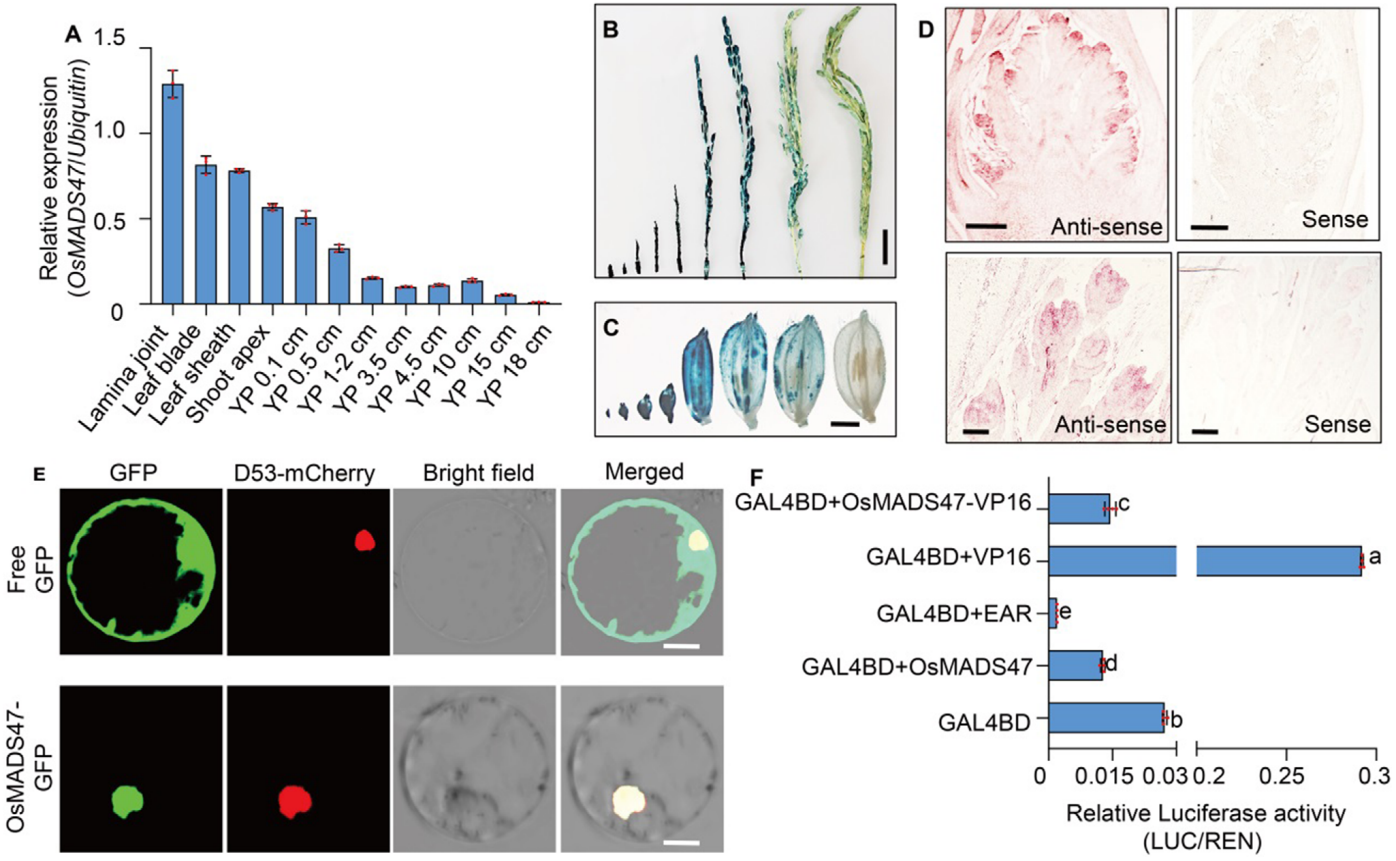
Expression pattern and transcriptional activity of OsMADS47. A) Analysis of *OsMADS47* expression in various organs by RT‐qPCR in NIP plants. Lamina joint, leaf blade, leaf sheath, and shoot apex were sampled at the vegetative stage. YP represents young panicles collected in different lengths. The *Ubiquitin* gene was used as an internal control (*n* = 3). B,C) GUS activity was detected in B) panicles, and C) spikelets at different developmental stages from *proOsMADS47:GUS* transgenic plants. Scale bars, B) 2 cm and C) 2 mm. D) In situ hybridization analysis of *OsMADS47* during panicle development in NIP. A sense probe was used as a negative control. Scale bar, 200 µm. E) Subcellular localization of OsMADS47 in rice protoplasts. D53‐mcherry was used as a nuclear marker. Scale bar, 10 µm. F) Transcriptional activity of OsMADS47 was tested in rice protoplasts using a GAL4/UAS‐based system. OsMADS47 was fused with GAL4 DNA‐binding domain (GAL4BD) driven by CaMV35S promoter. Four copies of the transcription repression domain EAR or transcription activation domain VP16 were fused with the OsMADS47 protein, respectively. The reporter construct is composed of a 35S minimal promoter with GAL4 UPSTREAM ACTIVATION SEQUENCE (UAS) driving Firefly luciferase (LUC) reporter gene. Renilla LUC (REN) was regarded as an internal control (*n* = 4). Different lowercase letters above bars indicate significant differences (p < 0.05) based on a one‐way ANOVA test.

To investigate expression pattern of the *OsMADS47* gene, we first performed quantitative RT‐PCR analysis. The expression of *OsMADS47* is relatively high during early stages of panicle development and gradually decreases as the panicle matures (**Figure** **3**A). We further generated transgenic rice plants expressing the *GUS* (*β‐glucuronidase*) reporter gene driven by the *OsMADS47* promoter and examined *OsMADS47* expression profile during spikelet development. Histochemical staining showed that the strongest signal was observed in the youngest panicles and spikelet hulls (Figure 3B,C). Furthermore, *OsMADS47* was strongly expressed in the primordia of rachis branches and spikelet meristems with a gradual decrease as development progressed (Figure 3D). Thus, *OsMADS47* is preferentially expressed in young tissues with high mitotic activity, supporting its role in the regulation of cell proliferation as presented above.

We next analyzed the subcellular localization of OsMADS47 and found that OsMADS47 colocalized in the nucleus with the nuclear marker D53‐mCherry in rice protoplasts^[^ ^40^ ^]^ (Figure 3E). We further investigated its transcriptional activity by a dual‐luciferase reporter (DLR) assay in rice protoplasts. As shown in Figure 3F, the relative luciferase activity in *GAL4BD‐OsMADS47* and *GAL4BD‐OsMADS47‐VP16* is obviously lower than that of the control constructs *GAL4BD* and *GAL4BD‐VP16*, respectively. These results showe that OsMADS47 has transcriptional repression activity.

### 2.4 OsMADS47 Directly Represses the Transcription of *GS3* and *GW8* to Regulate Rice Grain Shape

**Figure 4:**
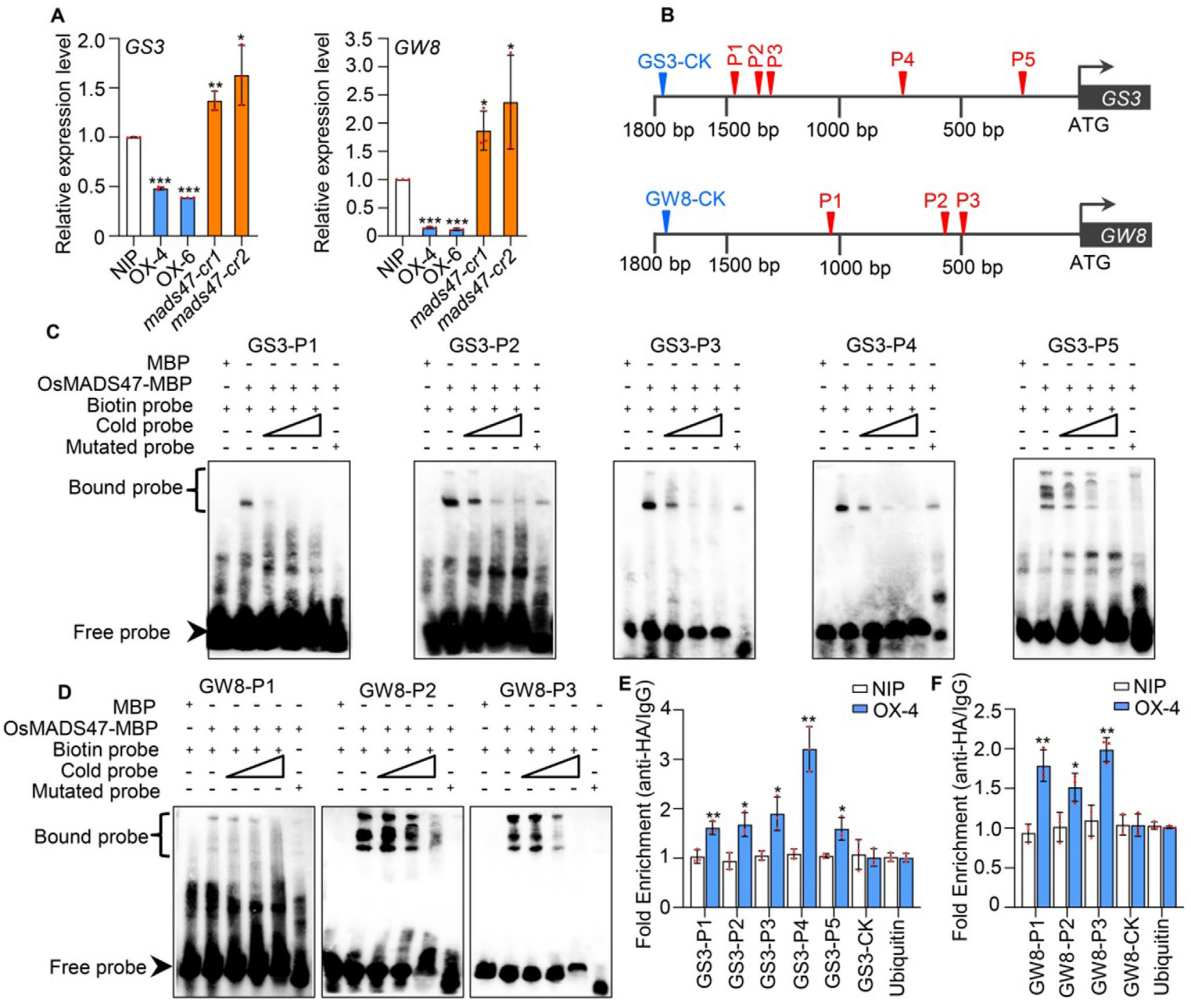
OsMADS47 negatively regulates the expression of *GS3* and *GW8*. A) Relative expression of *GS3* and *GW8* in young panicles of NIP and *OsMADS47* transgenic plants (*OX‐4*, *OX‐6*, *mads47‐cr1*, and *mads47‐cr2*). The *Ubiquitin* gene was used as an internal control (*n* = 3). B) Schematic of the *GS3* and *GW8* promoters. The sequence regions marked in the *GS3* promoter (P1‐P5) and *GW8* promoter (P1‐P3) indicate the tested regions containing the CArG boxes, respectively. GS3‐CK and GW8‐CK represent the negative control regions without the CArG boxes. C,D) EMSA of OsMADS47 with the DNA fragments containing the CArG boxes in the promoters of C) *GS3* and D) *GW8*. The MBP protein alone and the mutated probes were used as negative controls. The triangles indicate increasing amounts of unlabeled probes (10 times, 100 times, and 1 000 times of labeled probes) as competitors for OsMADS47 binding. E,F) ChIP‐qPCR analysis of OsMADS47 directly binding to the promoter regions of E) *GS3* and F) *GW8* in vivo using young panicles of *OsMADS47* transgenic plants (*OX‐4*). The numbers (GS3‐P1 to GS3‐P5 and GW8‐P1 to GW8‐P3) represent the primer pairs designed in the regions shown in (B). The enrichment of DNA fragments was first normalized to the input and then calculated as the fold changes in the immunoprecipitation sample with the anti‐HA antibody over that with the immunoglobulin G (IgG) (*n* = 3). The upstream regions of rice *Ubiquitin5* (*Ubiquitin*), *GS3* (GS3‐CK), and *GW8* (GW8‐CK) promoters were used as negative controls (*n* = 3). In A, E, and F, data are given as mean ± SD. Student's *t*‐test was used to generate the *p* values; *p < 0.05, **p < 0.01, ***p < 0.001.

In order to identify the downstream genes regulated by OsMADS47, we examined the transcript levels of some crucial genes regulating grain shape in young panicles of NIP, *OsMADS47* overexpression lines *OX‐4* and *OX‐6*, and *OsMADS47* knockout lines *mads47‐cr1* and *mads47‐cr2*. A couple of grain size‐related genes, such as *GL7*, *PPKL2*, and *SG1*, were significantly upregulated in the *OsMADS47* overexpression lines but obviously downregulated in the *OsMADS47* knockout lines compared with NIP, while *GS3* and *GW8* exhibited the opposite trend (**Figure** **4**A; Figure S5, Supporting Information). *GS3* and *GW8* have been reported to negatively regulate grain length and positively regulate grain width by influencing cell division of the spikelet hull, respectively.^[^ ^1^, ^2^ ^]^ Given that OsMADS47 has the transcriptional repression activity (Figure 3F), we speculated that *GS3* and *GW8* might be the direct target genes of OsMADS47. Electrophoresis mobility shift assay (EMSA) proved that MBP‐OsMADS47 could cause strong mobility shift of the biotin‐labeled probes (GS3‐P1 to GS3‐P5; GW8‐P1 to GW8‐P3) containing intact CArG box (the conserved MADS‐box‐binding site) from the *GS3* and *GW8* promoters, respectively, as compared with labeled probes containing mutated CArG box or the MBP protein alone (Figure 4B–D). This mobility shift could be weakened by nonlabeled competitive probes in a dose‐dependent manner (Figure 4C,D). Chromatin immunoprecipitation (ChIP)‐qPCR assay was further performed with anti‐HA antibody in young panicles of transgenic plants constitutively expressing *HA‐OsMADS47* (*OX‐4*). The fragments containing probe sequences (GS3‐P1 to GS3‐P5; GW8‐P1 to GW8‐P3) screened by EMSA of *GS3* and *GW8* promoters were obviously enriched in the chromatin fractions immunoprecipitated by anti‐HA antibody in *OX‐4* line compared with WT, while there was no significant enrichment in the other fragments (GS3‐CK, GW8‐CK, Ubiquitin) (Figure 4E,F). Together, these observations indicate that OsMADS47 can directly bind to the promoters of *GS3* and *GW8*, respectively.

**Figure 5:**
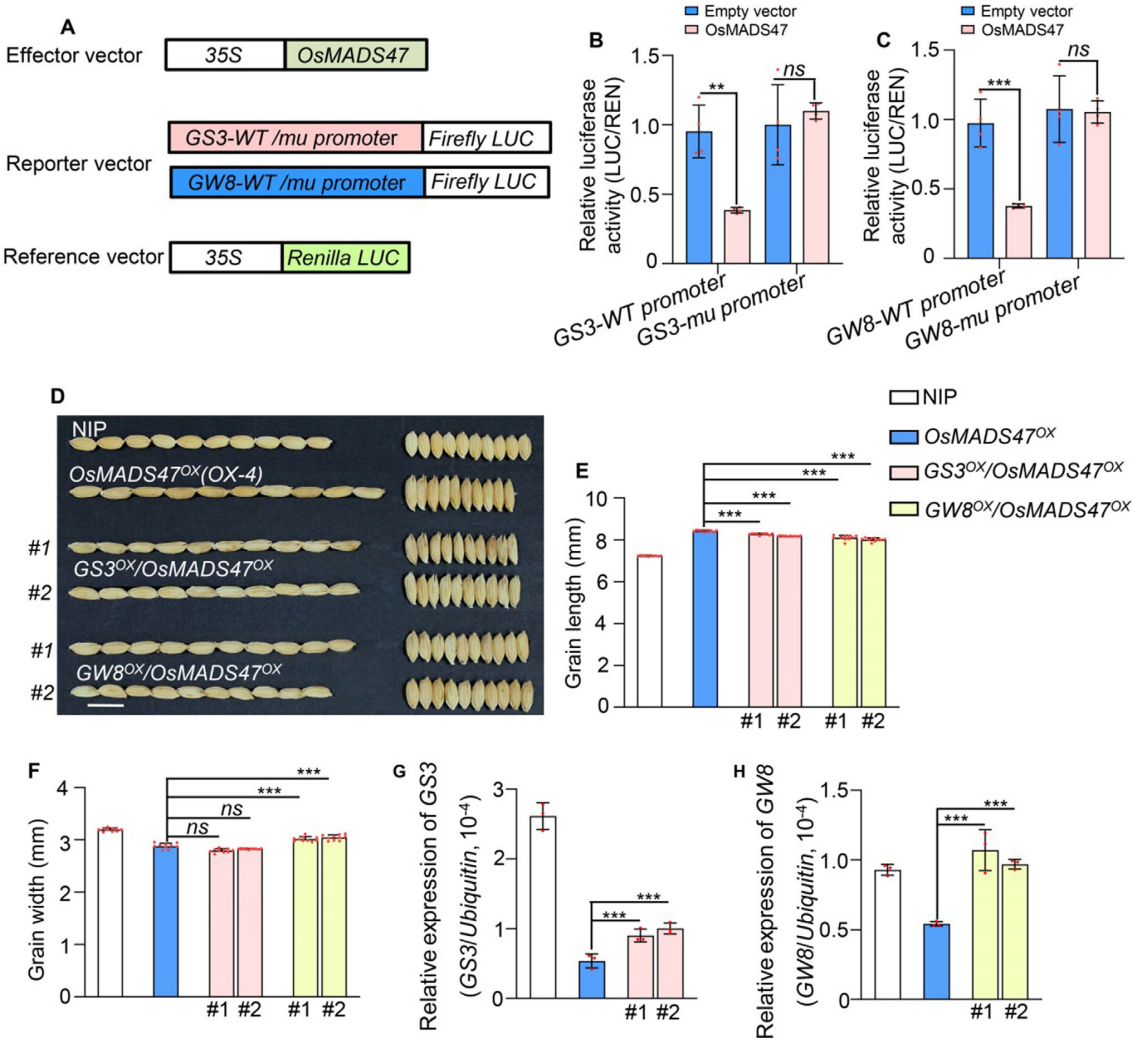
OsMADS47 represses the expression of *GS3* and *GW8* to regulate grain shape. A) Schematic representation of effector and reporter constructs used in dual luciferase reporter assays. The Firefly *luciferase* gene (*LUC*) driven by the wild type (WT) or mutated promoters of *GS3* and *GW8* were used as reporters, respectively. B,C) OsMADS47 represses the transcription of B) *GS3* and C) *GW8* in rice protoplasts. Relative activity was calculated as LUC/REN and normalized to the control co‐transfected with an empty effector construct and the corresponding reporter construct which was set to be one (*n* = 4). D) Grain morphology of NIP, *OsMADS47^OX^* (*OsMAD47* overexpression lines, *OX‐4* in Figure 1), *GS3^OX^/OsMADS47^OX^*, and *GW8^OX^/OsMADS47^OX^* plants. Scale bar, 1 cm. E,F) Grain length (E) and grain width (F) of NIP, *OsMADS47^OX^*, *GS3^OX^/OsMADS47^OX^* ^,^ and *GW8^OX^/OsMADS47^OX^* plants (*n* = 10). G,H) The relative expression of *GS3* (G) and *GW8* (H) in *OsMADS47^OX^*, *GS3^OX^/OsMADS47^OX^* ^,^ and *GW8^OX^/OsMADS47^OX^* plants, normalized to the rice *Ubiquitin* gene (*n* = 3). In B, C, and E–H, data are given as mean ± SD. Student's *t*‐test was used to generate the *p* values; *p < 0.05, **p < 0.01, ***p < 0.001; *ns*, no significant difference.

In order to examine the effect of OsMADS47 on the transcription of *GS3* and *GW8*, a transient co‐expression assay in rice protoplasts was carried out. The results showed that OsMADS47 significantly repressed LUC activity driven by the WT promoter of *GS3* or *GW8* compared with the empty effector, but had little effect on the LUC activity driven by the *GS3* or *GW8* promoter with all CArG boxes mutated (**Figure** **5**A–C). These results indicated that OsMADS47 represses the *GS3* and *GW8* gene expression via binding to the CArG boxes in their promoters.

Previous studies have shown that *GS3* overexpression resulted in a significant reduction in grain length and *GW8* overexpression made the grains short and wide compared with WT.^[^ ^2^, ^12^ ^]^ We postulated that *OsMADS47* overexpression (*MADS47^ox^*) lines showed increased grain length by repressing the expression of *GS3* and *GW8*. If we overexpress *GS3* and *GW8* in the *MADS47^ox^* lines, the grain length of *MADS47^ox^* lines should be reduced. Thus, we overexpressed *GS3* and *GW8* in the *OsMADS47* overexpression line (*OX‐4*). As expected, *GS3^OX^*/*OsMADS47^OX^* and *GW8^OX^*/*OsMADS47^OX^* transgenic plants formed shorter grains than *OsMADS47^OX^* plants (Figure 5D,E,G,H). Moreover, the grain of *GW8^OX^*/*OsMADS47^OX^* plants also became wider (Figure 5D,F,H). However, overexpression of *GS3* and *GW8* had no effect on the expression levels of *OsMADS47*, further indicating that slender grains of *OsMADS47^OX^* rely on the repressed expression of *GS3* and *GW8* (Figure S6, Supporting Information).

### 2.5 OsMADS47 Interacts with and Can Be Phosphorylated by OsMPK6

**Figure 6:**
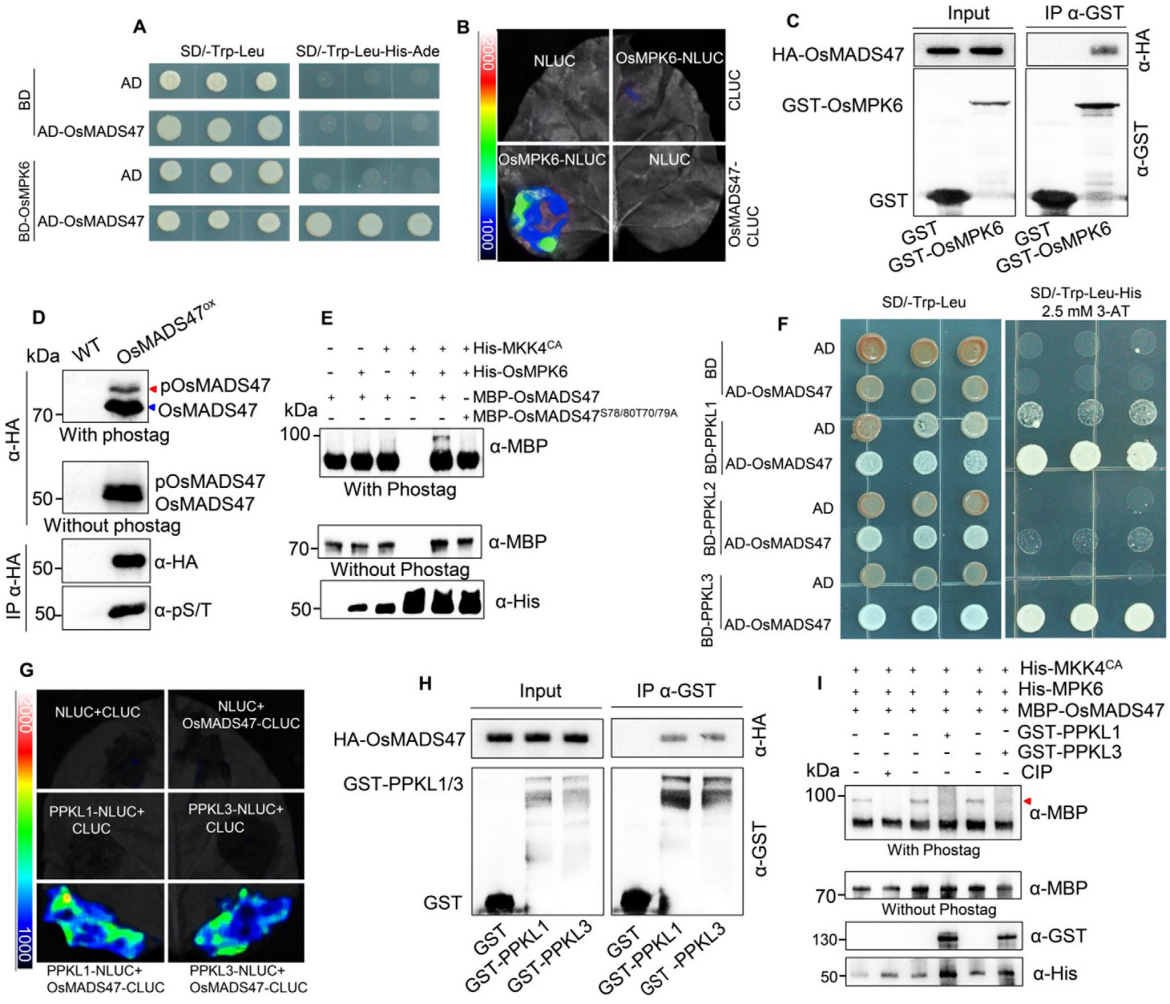
Phosphorylated OsMADS47 by OsMPK6 can be dephosphorylated by PPKL1 or PPKL3. A) OsMPK6 interacts with OsMADS47 in yeast cells. B) OsMPK6 associates with OsMADS47 in a split luciferase complementation assay in *N. benthamiana* leaves. C) OsMPK6 associates with OsMADS47 in a semi‐in vivo pull‐down assay. The protein extracts from *OsMADS47^OX^* (HA‐OsMADS47 overexpression) seedlings were pulled down with GST or GST‐MPK6 beads and detected using an anti‐HA antibody. D) The phosphorylation status of OsMADS47 in *OsMADS47^OX^* plants. Total proteins isolated from WT and *OsMADS47^OX^* (HA‐OsMADS47 overexpression) plants were separated on an SDS‐PAGE gel with or without Phos‐tag and detected with anti‐HA antibody, respectively. In addition, total protein was immunoprecipitated with anti‐HA‐specific agarose and the precipitates were detected with anti‐HA and anti‐Phospho Serine/Threonine (anti‐pS/T) antibodies, respectively. The red and blue arrows indicate phosphorylated and dephosphorylated OsMADS47, respectively. E) OsMPK6 mainly phosphorylates OsMADS47 on Thr‐70, Ser‐78, Thr‐79, Ser‐80 in vitro. The constitutively active form of OsMKK4 (His‐MKK4^CA^) was added to activate OsMKP6. F) PPKL1/2/3 physically interacts with OsMADS47 in yeast cells, respectively. G) PPKL1 or PPKL3 associates with OsMADS47 in a split luciferase complementation assay in *N. benthamiana* leaves. H) PPKL1 or PPKL3 associates with OsMADS47 in a semi‐in vivo pull‐down assay. The protein extracts from *OsMADS47^OX^* (HA‐OsMADS47 overexpression) seedlings were pulled down with GST, GST‐PPKL1, or GST‐PPKL3 beads and detected using an anti‐HA antibody. I) PPKL1 or PPKL3 dephosphorylates OsMADS47 in vitro. The phosphorylated OsMADS47 by OsMPK6 was used for the dephosphorylation reaction. The red arrow indicates phosphorylated MBP‐OsMADS47. The calf intestinal alkaline phosphatase (CIP) was used as a positive control.

To further explore the molecular mechanism by which OsMADS47 regulates rice grain shape, we performed a yeast two‐hybrid assay to identify its interacting partners. One of the interactors obtained was the mitogen‐activated protein kinase OsMAPK6 (OsMPK6), which positively regulates grain size and weight in rice^[^ ^27^ ^]^ (**Figure** **6**A). Strong luminescence was observed when OsMADS47‐CLUC was co‐expressed with OsMPK6‐NLUC in *N. benthamiana* leaves, while no signals were detected in the combinations OsMADS47‐CLUC/NLUC and CLUC/OsMPK6‐NLUC (Figure 6B). The association of OsMADS47 with OsMPK6 was further demonstrated by semi‐in vivo pull‐down assay, in which the GST‐OsMPK6 protein could pull down the HA‐OsMADS47 protein from the total protein extracts of the *OsMADS47^ox^* seedlings (Figure 6C). Thus, these results confirm that OsMADS47 interacts with OsMPK6 in vivo.

As OsMPK6 acts as a protein kinase, we tested whether OsMADS47 was phosphorylated in vivo. Protein extracts from young panicles of the *OsMADS47* overexpression line (*OX‐4*) were separated using Phos‐tag and standard SDS‐PAGE, respectively. Immunoblotting with anti‐HA antibody showed an obvious mobility shift in the Phos‐tag gel, compared with the standard SDS‐PAGE gel, representing phosphorylated OsMADS47 (Figure 6D, upper panel). Protein fractions immunoprecipitated by anti‐HA beads were further subjected to detection by the anti‐phospho Ser/Thr antibody after separation on the standard SDS‐PAGE gel. A strong signal was detected at the position of HA‐OsMADS47 indicated by the anti‐HA antibody (Figure 6D, lower panel), suggesting that phosphorylation occurs at the Serine/Threonine residues of OsMADS47. To find out the exact residues modified by phosphorylation, the shifted band detected on the Phos‐tag gel was then subjected to liquid chromatography‐tandem mass spectrometry (LC‐MS/MS) analysis. This assay revealed four potential phosphorylation sites within OsMADS47, including Thr‐70, Ser‐78, Thr‐79, Ser‐80 (Figure S7, Supporting Information).

To confirm these phosphorylation sites, we mutated Thr‐70, Ser‐78, Thr‐79, Ser‐80 to alanine (OsMADS47^S78/80T70/79A^) and then evaluated their effects on OsMPK6 phosphorylation using in vitro kinase assay. As shown in Figure 6E, we detected an obvious mobility shift corresponding to phospho‐MBP‐OsMADS47 in the Phos‐tag SDS‐PAGE gel after adding His‐OsMPK6, which was phosphorylated and activated by OsMKK4^CA^, a constitutively activated version of OsMKK4 that carries Thr238Asp and Ser244Asp mutations.^[^ ^41^ ^]^ By contrast, the mutant protein MBP‐OsMADS47^S78/80T70/79A^ could not be phosphorylated by OsMPK6 (Figure 6E), indicating that these four sites are the major sites where OsMPK6 phosphorylates OsMADS47. Altogether, these results suggest that OsMADS47 can interact with and be phosphorylated by OsMPK6 in vitro and in vivo. To examine whether these phosphorylation sites are essential for the regulation of grain shape in rice, we overexpressed the OsMADS47^S78/80T70/79A^ mutant gene driven by the rice *Actin1* promoter in the NIP background. *ProActin:OsMADS47^S78/80T70/79A^* transgenic plants exhibited no significant difference in grain length and grain width compared to WT (Figure S8, Supporting Information), implying that the lack of phosphorylation due to S/T‐to‐A mutations at the 70, 78, 79 and 80th residues impaired the functionality of OsMADS47 in the regulation of grain shape.

### 2.6 Phosphorylated OsMADS47 Can Be Dephosphorylated by PPKL1/3

Using the yeast two‐hybrid assay, we found strong interactions between OsMADS47 and PPKL1/3 on the selection medium SD/‐Trp‐Leu‐His plus 2.5 mM 3‐AT. In contrast, OsMASD47 showed a very weak interaction with PPKL2 under the same selection condition (Figure 6F) although it could be detected without adding 3‐AT (Figure S9B, Supporting Information). Interestingly, we found that the phosphorylation status of OsMADS47 affects its interaction with PPKL1/2/3. PPKL1/3 had stronger interaction with the phosphorylation‐mimicking OsMADS47^S78/80T70/79D^ mutant than the dephosphorylation‐mimicking OsMADS47^S78/80T70/79A^ mutant and OsMADS47 (Figure S9A,C, Supporting Information). Conversely, PPKL2 had stronger interaction with OsMADS47^S78/80T70/79A^ and OsMADS47 than OsMADS47^S78/80T70/79D^ (Figure S9B, Supporting Information). Moreover, compared with the upregulation of *PPKL2* in the *OsMADS47* overexpression lines (Figure S5, Supporting Information), the expression level of *PPKL1/3* remained unchanged, suggesting that the transcription of *PPKL1/3* is not regulated by OsMADS47 (Figure S10, Supporting Information). Previous studies have shown that overexpression of *PPKL1/3* resulted in short grains, whereas the overexpression of *OsPPKL2* produced long grains.^[^ ^42^ ^]^ Our results here further support that these three homologous proteins PPKL1/2/3 play diversified roles in regulating grain size.

We next tested whether PPKL1/3 interact with OsMADS47 in vivo. Strong luminescence was observed when OsMADS47‐CLUC was co‐expressed with PPKL1‐NLUC or PPKL3‐NLUC, whereas there was no signal in the combinations PPKL1/3‐NLUC/CLUC and NLUC/OsMADS47‐CLUC (Figure 6G). Semi‐in vivo pull‐down assay showed that GST‐PPKL1/3 could pull down HA‐OsMADS47 from the rice seedling extracts (Figure 6H). Altogether, these results suggest that PPKL1/3 directly interact with OsMADS47.

PPKL1/3 are known as protein Ser/Thr phosphatase with kelch‐like repeat domains.^[^ ^4^ ^]^ Thus, we performed an in vitro dephosphorylation assay to determine whether PPKL1/3 can dephosphorylate OsMADS47. MBP‐OsMADS47 was phosphorylated by His‐OsMPK6 beforehand as described above (Figure 6E). The phosphorylated MBP‐OsMADS47 was then used in a dephosphorylation reaction with GST‐PPKL1/3. The reaction mixture was subjected to separation on Phos‐tag and standard SDS‐PAGE gel, respectively. As shown in the Phos‐tag SDS‐PAGE gel in Figure 6I, the shifted band representing phosphorylated MBP‐OsMADS47 triggered by His‐OsMPK6 disappeared after treatment with GST‐PPKL1/3 or the positive control calf intestinal alkaline phosphatase (CIP), suggesting that PPKL1/3 can dephosphorylate OsMADS47 in vitro.

### 2.7 Phosphorylation Status of OsMADS47 Affects Its Stability and Transcription Repression Activities on Target Genes

**Figure 7:**
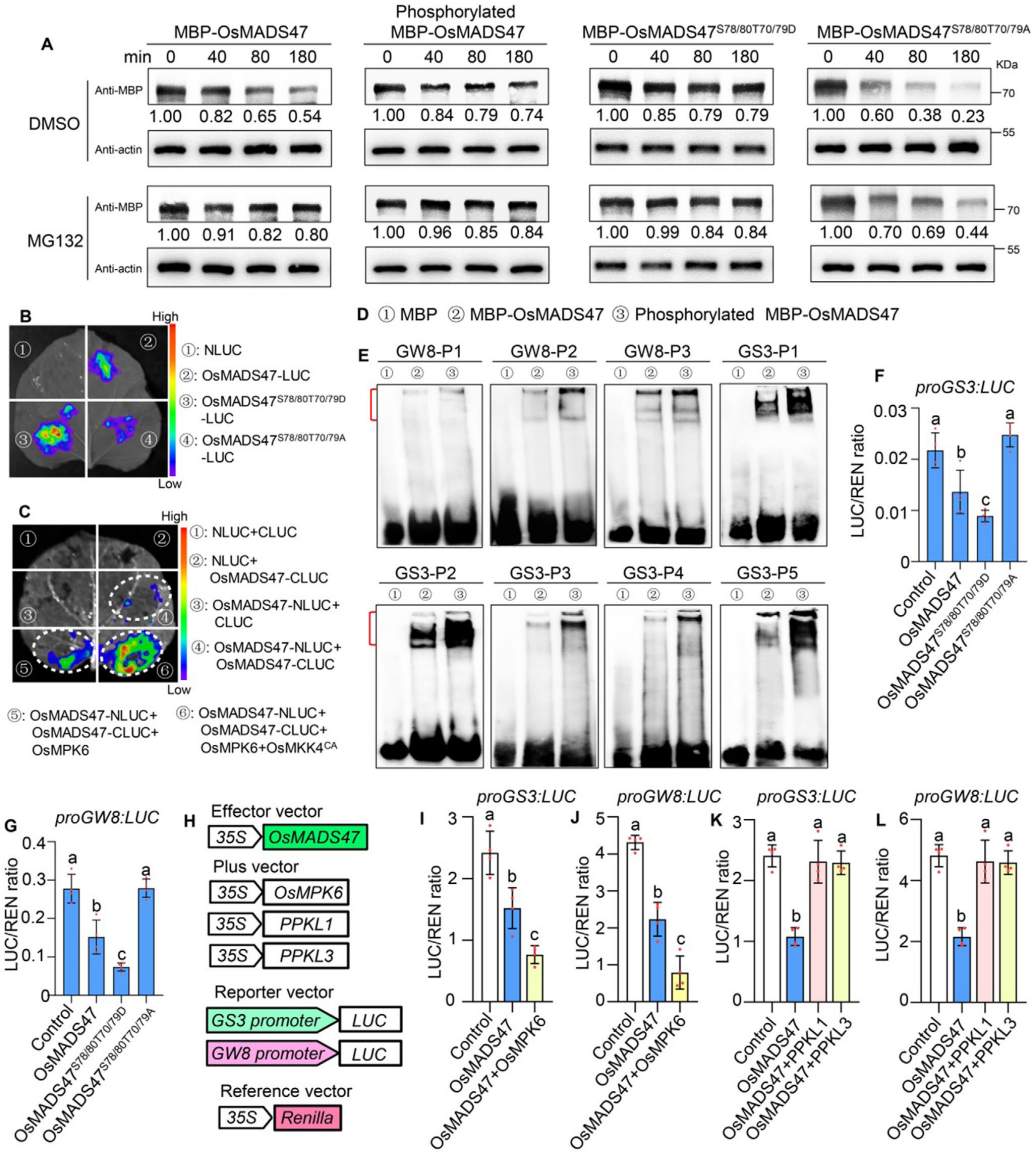
Phosphorylation status of OsMADS47 affects its stability and transcription repression activities on downstream genes *GS3* and *GW8*. A) The phosphorylation status of OsMADS47 affects its stability in a cell‐free degradation system with or without 50 µm MG132. Equal amounts of total proteins extracted from NIP seedlings are incubated with MBP‐OsMADS47, phosphorylated MBP‐OsMADS47 by OsMPK6, MBP‐OsMADS47^S78/80T70/79D^ and MBP‐OsMADS47^S78/80T70/79A^ in the presence of ATP. Recombinant proteins of OsMADS47 were detected with an anti‐MBP antibody. Numbers between the bands indicate the relative OsMADS47 protein levels after normalization with actin. Three independent replicates show similar results. B) Phosphorylation status of OsMADS47 affects its stability in transient expression assay of luciferase activity in *N. benthamiana* leaves. C) OsMPK6 and OsMKK4^CA^ affect the dimerization of OsMADS47 in transient expression assay of luciferase activity in *N. benthamiana* leaves. D,E) The DNA‐binding activities of MBP‐OsMADS47 and phosphorylated MBP‐OsMADS47 to probes containing the CArG boxes from on the *GS3* and *GW8* promoters as shown in Figure 4B. Red parentheses indicate the bound probes. F,G) Phosphorylation status of OsMADS47 affects its repression activities on *GS3* and *GW8* promoters in rice protoplasts. The effector and reporter constructs used are shown in Figure 5A. H) Schematic representation of constructs used in dual luciferase reporter assay. I–L) The effect of OsMPK6 (I,J), PPKL1/3 (K,L) on the transcriptional repression activities of OsMADS47 on the *GS3* (I,K) and *GW8* (J,L) expression (*n* = 4). In F‐G, and I‐L, different lowercase letters above bars indicate significant differences (*p* < 0.05) based on a one‐way ANOVA test.

As protein phosphorylation is closely related to its stability,^[^ ^43^ ^]^ we next tested whether the stability of the OsMADS47 protein was affected by its phosphorylation status using a cell‐free degradation assay. The results revealed that phosphorylated MBP‐OsMADS47 by OsMPK6 and the phosphorylation‐mimicking MBP‐OsMADS47^S78/80T70/79D^ degraded more slowly than MBP‐OsMADS47, while the dephosphorylation‐mimicking MBP‐OsMADS47^S78/80T70/79A^ degraded the fastest (**Figure** **7**A). On the other hand, degradation of MBP‐OsMAD47 and MBP‐OsMADS47^S78/80T70/79A^ was blocked by MG132, implying that OsMADS47 degradation is mediated by the 26S proteasome system (Figure 7A). Furthermore, we performed transient expression assays in *N. benthamiana* leaves and rice protoplasts. In both systems, MBP‐OsMADS47^S78/80T70/79D^ was shown to be more stable than OsMADS47^S78/80T70/79A^ and OsMADS47 (Figure 7B; Figure S11, Supporting Information). Consistent with the phosphorylation‐dependent stability of OsMADS47, the phosphorylation‐mimicking OsMADS47^S78/80T70/79D^ exhibited stronger homodimerization ability than OsMADS47 in the yeast (Figure S12, Supporting Information). In contrast, dephosphorylation‐mimicking MBP‐OsMADS47^S78/80T70/79A^ failed to self‐interact (Figure S12, Supporting Information). We further demonstrated that OsMADS47 homodimerization in *N. benthamiana* leaves was enhanced by OsMPK6 alone and further potentiated by the OsMKK4^CA^‐OsMPK6 cascade (Figure 7C). EMSA assays revealed that phosphorylated MBP‐OsMADS47 also exhibited stronger DNA‐binding activities on probes containing the CArG box from the *GS3* and *GW8* promoters, compared with MBP‐OsMADS47 (Figure 7D,E).

To test whether the interactions of OsMPK6 and PPKL1/3 with OsMADS47 have effects on the expression of its downstream genes *GS3* and *GW8*, we performed transient dual‐luciferase (LUC) assay in rice protoplasts. The results showed that OsMADS47^S78/80T70/79D^ is the strongest suppressor and OsMADS47^S78/80T70/79A^ is the weakest one on the expression of *GS3* and *GW8* (Figure 7F,G). Consistent with this, when *OsMPK6* was coexpressed with *OsMADS47*, the OsMADS47‐dependent inhibition of LUC activity was further enhanced in both *GS3pro:LUC* and *GW8pro:LUC* reporters, whereas when *PPKL1/3* was coexpressed with *OsMADS47*, the OsMADS47‐dependent inhibition of LUC activity was significantly attenuated in both reporters, implying that the transcriptional repression activity of OsMADS47 on *GS3* and *GW8* is indeed modulated by OsMPK6 and PPKL1/3 (Figure 7H–L). In addition, the transcription levels of *GS3* and *GW8* were significantly up‐regulated in young panicles of *OsMPK6^RNAi^*/*OsMADS47^OX^* and *PPKL1/3^OX^*/*OsMADS47^OX^* plants (Figure S13, Supporting Information), suggesting that dephosphorylation of OsMADS47 facilitates the transcription of downstream genes.

### 2.8 Genetic Analysis of *OsMPK6*, *OsMADS47*, and *PPKL1/3*

**Figure 8:**
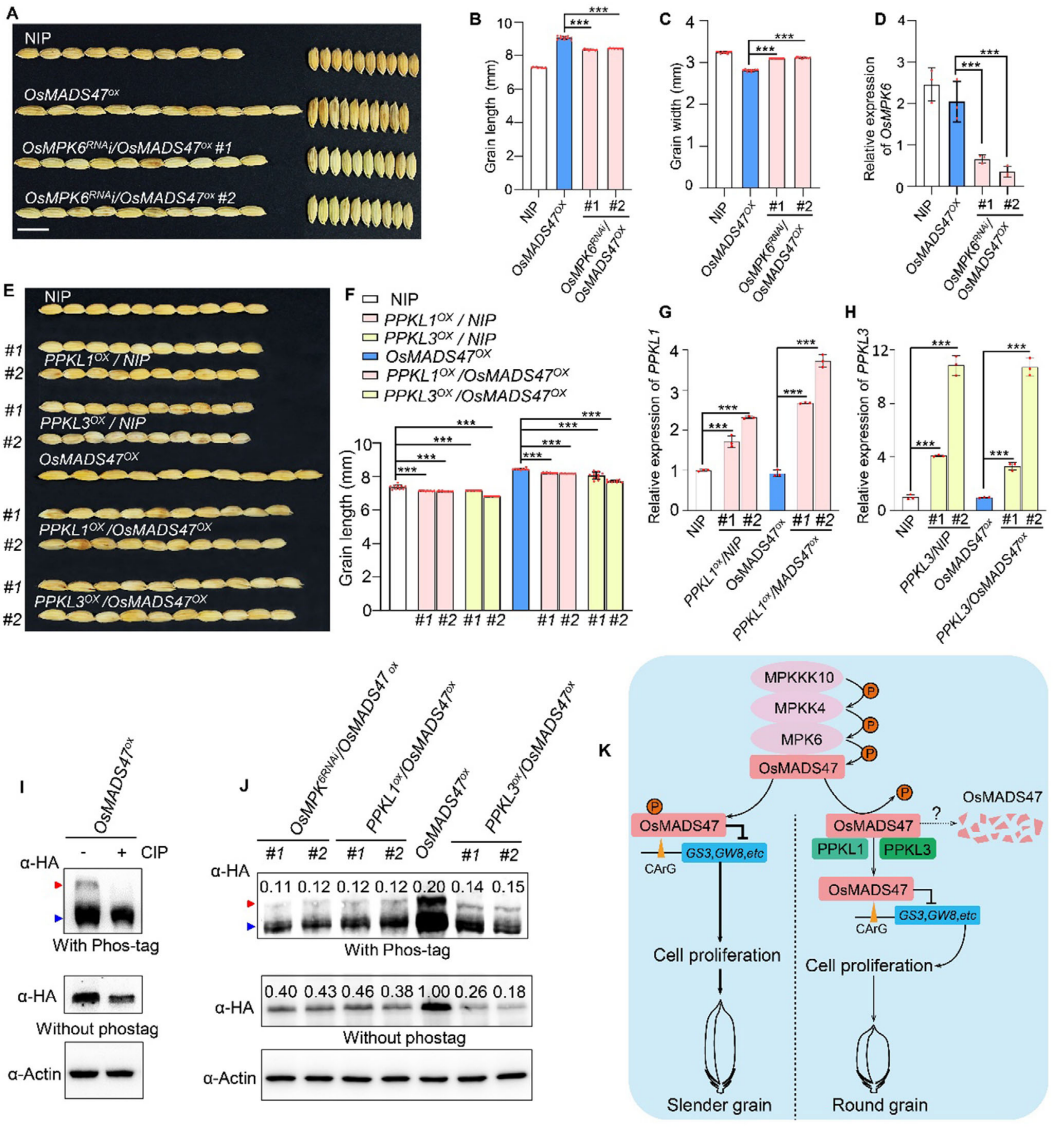
Genetic analyses of *OsMADS47*, *OsMPK6*, *PPKL1*, and *PPKL3*. A) Grain morphology of NIP, *OsMADS47^OX^*, and *OsMPK6^RNAi^*/*OsMADS47^OX^* plants. Scale bar, 1 cm. B) Grain length and C) grain width of NIP, *OsMADS47^OX^* and *OsMPK6^RNAi^/OsMADS47^OX^* plants (*n* = 10). D) Relative expression of *OsMPK6* in NIP, *OsMADS47^OX^* and *OsMPK6RNAi/OsMADS47^OX^* plants. E) Grain morphology of NIP, *OsMADS47^OX^*, *PPKL1*‐overexpression plants in NIP or *OsMADS47^OX^* background (*PPKL1^OX^*/NIP, *PPKL1^OX^*/*OsMADS47^OX^*) and *PPKL3*‐overexpression plants in NIP or *OsMADS47^OX^* background (*PPKL3^OX^*/NIP, *PPKL3^OX^*/*OsMADS47^OX^*). Scale bars, 1 cm. F) Grain length of NIP, *OsMADS47^OX^*, *PPKL1*‐overexpression and *PPKL3*‐overexpression plants (*n* = 10). (G,H) Relative expression of G) *PPKL1* and H) *PPKL3* in NIP, *OsMADS47^ox^*, *PPKL1*‐overexpression (*PPKL1^OX^*/NIP, *PPKL1^OX^*/*OsMADS47^OX^*) and *PPKL3*‐overexpression (*PPKL3^OX^*/NIP, *PPKL3^OX^*/*OsMADS47^OX^*) plants, respectively. In D, G and H, data are given as mean ± SD. Student's *t*‐test was used to generate the *p* values; ***p < 0.001. The *Ubiquitin* gene was used as an internal control (*n* = 3). I) Phosphorylated OsMADS47 in *OsMADS47^OX^* plant was digested by the calf intestinal alkaline phosphatase (CIP). J) The phosphorylated and total protein levels of OsMADS47 in *OsMADS47^OX^* and *OsMPK6^RNAi^/OsMADS47^OX^*, *PPKL1^OX^*/*OsMADS47^OX^* and *PPKL3^OX^*/*OsMADS47^OX^* plants. Numbers above the bands in the upper panel indicate the ratio of phosphorylated fraction in total OsMADS47 protein. Numbers above the bands in the middle panel indicate the relative OsMADS47 protein levels after normalization with actin. The red and blue arrows indicate phosphorylated and non‐phosphorylated OsMADS47, respectively, in I and J. K) A proposed working model for OsMADS47 in the regulation of rice grain shape. OsMADS47 acts downstream of the OsMKKK10‐OsMKK4‐OsMPK6 cascade pathway and can be phosphorylated by OsMPK6. Phosphorylated OsMADS47 not only becomes stable, but also enhances its transcription repression activities on *GS3* and *GW8*, resulting in long and narrow (slender) grains. PPKL1 or PPKL3 can dephosphorylate OsMADS47 to balance its phosphorylation level in rice, releasing the expression of *GS3* and *GW8* which leads to short and wide (round) grains.

Given that OsMPK6 and PPKL1/3 interact with and phosphorylate or dephosphorylate OsMADS47, all of which have been previously reported or revealed in this study to regulate grain shape by influencing cell proliferation, we attempted to reveal the genetic relationship among *OsMPK6*, *PPKL1/3*, and *OsMADS47*.^[^ ^4^, ^32^, ^44^ ^]^ We hypothesized that the increased grain length of *OsMADS47* overexpression (*MADS47^ox^* ^)^ lines is dependent on the phosphorylation status of OsMADS47, which is subjected to the regulation by OsMPK6 and PPKL1/3. To test this, we downregulated the *OsMPK6* expression through RNA interference (RNAi) technology or overexpressed *OsPPKL1/3* driven by the CaMS35S promoter in the *MADS47^ox^* lines (*OX‐4*). As shown in **Figure** **8**D, two representative lines *OsMPK6^RNAi^‐1/OsMADS47^OX^* and *OsMPK6^RNAi^‐2/OsMADS47^OX^* showed obvious reductions in *OsMPK6* expression. Statistical analysis showed that grain length of *OsMPK6^RNAi^‐1*/*OsMADS47^OX^* and *OsMPK6^RNAi^‐2*/*OsMADS47^OX^* decreased by ≈6.9% and 7.8%, respectively, compared with that of *OsMADS47^OX^*, while grain width of both lines increased by ≈10.8% and 10.0%, respectively (Figure 8A–C), indicating that the slender‐grain phenotype in *OsMADS47* overexpression plants depends on the OsMPK6 function. PPKL1/3 have been reported to negatively regulate rice grain length.^[^ ^33^ ^]^ Consistent with previous studies, overexpression of *PPKL1* and *PPKL3* in NIP reduced grain length by ≈～0.9% and 3.4%, respectively(Figure 8E–H). Similarly, overexpression of *PPKL1* and *PPKL3* in the *OsMADS47^OX^* background also significantly reduced grain length compared to *OsMADS47^OX^* (Figure 8E–H). These results suggested that the positive regulation of grain length by OsMADS47 is restrained by the function of PPKL1/3.

Considering that OsMPK6 and PPKL1/3 function as protein kinase and phosphatase, respectively, we analyzed the phosphorylation level of OsMADS47 in different transgenic plants, i.e., *OsMPK6^RNAi^*/*OsMADS47^OX^*, *PPKL1/3^OX^*/*OsMADS47^OX^* and *OsMADS47^OX^*. The shifted band of phosphorylated HA‐OsMADS47 in *OsMADS47^OX^* plants was abolished after treatment with calf‐intestinal alkaline phosphatase (CIP), confirming that it is the phosphorylated form of OsMADS47 in rice (Figure 8I). The phosphorylation levels of OsMADS47 in *OsMPK6^RNAi^*/*OsMADS47^OX^* and *PPKL1/3^OX^*/*OsMADS47^OX^* plants are obviously lower than that in *OsMADS47^OX^* plants, implying that phosphorylation status of OsMADS47 is fine‐tuned by OsMPK6 and PPKL1/3 in rice plant (Figure 8J, upper panel). Meanwhile, the total protein levels of OsMADS47 were significantly reduced in *OsMPK6^RNAi^*/*OsMADS47^OX^* and *PPKL1/3^OX^*/*OsMADS47^OX^* plants, compared with that in *OsMADS47^OX^* plants (Figure 8J, middle panel), although the transcript levels of *OsMADS47* were not affected by *OsMPK6* RNAi or *PPKL1/3* overexpression (Figure S6, Supporting Information). These results are consistent with the findings from cell‐free degradation assay and transient expression analysis in *N. benthamiana* leaves and rice protoplasts (Figure 7A,B; Figure S11, Supporting Information), demonstrating that OsMADS47 stability depends on its phosphorylation status in rice plant.

## 3 Discussion

Grain shape is an important agronomic trait for rice yield and appearance quality.^[^ ^43^ ^]^ Although a large number of genes controlling rice grain shape have been cloned, our understanding of the regulatory pathways of grain shape is still fragmentary. In this study, we identified a hub gene *OsMADS47* which crosslinked several grain shape‐related pathways by interacting with *OsMPK6*, *PPKL1/3*, *GS3*, and *GW8*. OsMPK6 together with OsMKKK10 and OsMKK4 functions as a signaling cascade to promote grain growth by increasing cell proliferation.^[^ ^27^ ^]^ In rice, BRs play an important role in the regulation of grain size, leaf angle and yield potential.^[^ ^45^ ^]^ However, the possible factors integrating MAPK signaling and BR pathway are as yet unknown. Here, we found that OsMADS47 can be dephosphorylated by PPKL1/3 which share similarity with Arabidopsis BSU1 and BSL1.^[^ ^32^ ^]^ In addition, OsMADS47 has been reported as a negative regulator of BR response.^[^ ^46^ ^]^ However, unlike other BR‐insensitive mutants with small and round grains, *OsMADS47* overexpression plants exhibit long and slender grains (Figure 1). Similarly, the *DWARF and LOW TILLERING* (*DLT*)/*D62*/*GS6* gene encoding a GRAS family TF negatively regulates grain size while positively regulating BR signaling and gibberellic acid (GA) metabolism.^[^ ^47^, ^48^ ^]^ G‐protein signaling plays vital roles in diverse growth and developmental processes in plants.^[^ ^49^ ^]^ Here, we further demonstrate that *OsMADS47* integrates G‐protein and BR signaling in grain shape regulation via directly modulating the expression level of *GS3* encoding a Gγ subunit in rice.^[^ ^50^ ^]^

OsMADS47 has a characteristic modular structure (M‐I‐K‐C) which consists of the MADS‐box (M), intervening (I), keratin‐like (K), and C‐terminal (C) domain from the N‐ to C‐terminus (Figure S7B, Supporting Information).^[^ ^51^ ^]^ The MADS domain mediates both DNA binding and protein dimerization,^[^ ^52^ ^]^ while the I domain contributes to DNA‐binding specificity.^[^ ^53^ ^]^ We identified four conserved phosphorylation sites (Thr‐70, Ser‐78, Thr‐79, and Ser‐80) located at the end of MADS‐box domain and the I domain, respectively (Figure S7, Supporting Information). Sequence alignment of OsMADS47 with its orthologs revealed complete conservation of Thr‐70, Ser‐78, and Ser‐80 phosphorylation sites across monocots and dicots, with Thr‐79 showing only conservative Ser substitutions in some species while still retaining phosphorylation potential, indicating these phosphorylation sites play essential roles in OsMADS47 function. (Figure S14, Supporting Information). Since MADS TFs typically function through homo‐/hetero‐dimerization,^[^ ^54^, ^55^ ^]^ our results demonstrate that phosphorylation at these sites significantly enhances OsMADS47 dimerization (Figure 7C; Figure S12, Supporting Information). Consistent with enhanced dimerization, phospho‐activated OsMADS47 showed stronger binding ability to CArG‐box motifs in the promoters of *GS3* and *GW8*, forming homodimers and higher‐order quartet complexes (Figure 7D,E). These results suggest that phosphorylation of OsMADS47 may regulate its transcriptional activity by modulating its oligomeric state.

OsMPK6 phosphorylates OsMADS47 and *OsMPK6^RNAi^/OsMADS47^OX^* exhibited wider grains than *OsMADS47^OX^* (Figure 8A,C). However, a previous study showed that knockdown of the *OsMPK6* gene expression by RNAi in the wild type background significantly decreased the grain length without significant change in grain width.^[^ ^27^ ^]^ The different effects of *OsMPK6* RNAi on grain width may be explained by their genetic background. In the *OsMADS47* overexpression background, expression of many genes related to grain width was significantly altered, compared with WT. E.g., the positive regulator of grain width, GW8, and its downstream gene *GL7* were down‐regulated and up‐regulated, respectively, in *MADS47^ox^* plants (Figure 4A; Figure S5, Supporting Information). In the *OsMPK6^RNAi^/OsMADS47^OX^* lines, the decreased expression of *OsMPK6* effectively upregulated the expression of *GW8* which is maintained at a relatively low level due to the strong transcriptional suppression by OsMADS47 in the *OsMADS47^OX^* lines (Figure S13B, Supporting Information). However, the upregulation of *GW8* may not be so effective in the *OsMPK6^RNAi^* lines as the expression level of the *GW8* and other genes is maintained at normal levels. However, the slender‐grain phenotype of *OsMADS47^OX^* was not completely suppressed by knockdown of *OsMPK6* (Figure 8A‐D). In Arabidopsis, two well‐known MAPKs MPK3 and MPK6 play redundant roles in many aspects of plant growth and development by phosphorylating downstream substrates which specify the function of MAPK cascades.^[^ ^56^ ^]^ In our study, we found that OsMADS47 also physically interacts with OsMPK3 in yeast cells, implying that OsMADS47 might associate with other MAPKs to regulate grain shape (Figure S15, Supporting Information).

Based on these findings, we propose a working model in which OsMADS47 acts as a central regulatory hub to coordinate phosphorylation‐dependent signaling for grain shape determination (Figure 8K). During grain development, the OsMKKK10‐OsMKK4‐OsMPK6 MAP kinase cascade is activated by certain upstream signals (to be identified). This signaling cascade subsequently phosphorylates OsMADS47, thereby enhancing its transcriptional repression activity toward two key negative regulators of grain elongation—GS3 and GW8. Reduced expression of *GS3* and *GW8* promotes longitudinal cell proliferation in spikelet hulls, leading to slender grain formation. Concurrently, PPKL1/3 protein phosphatases counteract this process by dephosphorylating OsMADS47, weakening its repressive function. This elevates *GS3* and *GW8* expression, shifting the cellular growth pattern toward lateral expansion and resulting in round grains. The antagonistic actions of the OsMPK6 kinase and PPKL1/3 phosphatases establish a dynamic phosphorylation equilibrium of OsMADS47, which fine‐tunes the expression levels of *GS3* and *GW8*. This balance governs cell proliferation geometry in developing spikelet hulls, ultimately determining final grain morphology.

Overexpression of *OsMADS47* resulted in slender grains with significant increase in grain length and moderate decrease in grain width, transforming the round grain of the japonica rice variety Nipponbare to the indica rice‐like slender grain (Figure 1A–D). Given the dramatic changes in grain shape caused by overexpression of *OsMADS47*, we claimed that OsMADS47 controls grain shape as a positive regulator of grain length and negative regulator of grain width. Intriguingly, the knockout lines of *MADS47* also showed a decrease in grain width besides reduced grain length (Figure 1N–P). This contradictory effect on grain width may be explained by severe defects in the overall development of the Os*MADS47* knockout lines such as reduced plant height, smaller leaf and panicle size, lower fertility etc. (Figure S3, Supporting Information). This explanation is plausible as MADS‐box gene family function in many aspects of plant growth and development, including flowering time control, meristem identity, floral organ identity, as well as development of vegetative organs such as root and leaf.^[^ ^52^ ^]^ The contradictory effects on grain shape have also been reported for *OsMADS1*, the most well‐characterized member of the rice MADS‐box gene family. OsMADS1*^lgy3^*, an alternatively spliced protein of OsMADS1, is shown to be associated with formation of long and slender grains with high breeding application value.^[^ ^3^ ^]^ However, loss‐of‐function of *OsMADS1* leads to small spikelet with reiterative formation of glume or extra spikelet formation within spikelet and defective organ identity.^[^ ^57^ ^]^

It is worth noting that not all the *OsMADS47* overexpression lines showed increased grain yield. This variation in grain yield can be attributed to the different expression levels of *OsMADS47* in these transgenic lines. Interestingly, only class II with moderate overexpression of *OsMADS47* showed a significant increase in grain yield per plant due to increased spikelet number per panicle with slight negative effects on the TGW. Ironically, class III with extremely high overexpression of *OsMADS47* showed a decreased grain yield due to the reduced grain weight and poor seed setting rate (Figure S1, Supporting Information). To elucidate the molecular basis of these phenotypic changes, we analyzed the expression of panicle architecture‐related genes and hormone pathway‐related genes in *OsMADS47* overexpressing lines.^[^ ^58^, ^59^ ^]^ Notably, the negative regulator of panicle size *FON1* was significantly downregulated while the positive regulator *IPA1* was upregulated in the *OsMADS47* overexpression plants (Figure S16A, Supporting Information). Furthermore, auxin signaling components, including some members of *Aux/IAA* and *YUCCA* families, were markedly suppressed, whereas cytokinin‐related genes (type‐A response regulators*, OsCKX2, DST*) remained unaffected (Figure S16B–D, Supporting Information). Intriguingly, these results contrast with the established role of the OsMKKK10‐OsMKK4‐OsMPK6 cascade in panicle development, which directly regulates cytokinin homeostasis via phosphorylation of DST to activate *OsCKX2* expression, thereby balancing grain number and size.^[^ ^30^ ^]^ Our findings suggest that OsMADS47 modulates panicle architecture primarily through auxin signaling dynamics and direct transcriptional regulation of key developmental regulators such as *IPA1* and *FON1*, rather than cytokinin‐dependent pathways.

This auxin‐mediated regulation may also indirectly influence grain development, as auxin signaling is known to crosstalk with pathways controlling grain size and filling. For instance, the ROP GTPase OsRac1 was reported to phosphorylate and activate OsMPK6 to increase grain size and accelerate grain filling.^[^ ^28^ ^]^ The observed reduction in TGW and seed setting rate in class III lines could thus reflect an auxin signaling imbalance under extreme *OsMADS47* overexpression, disrupting the coordination between panicle branching and grain maturation. Together, these results imply that OsMADS47, as a critical node linking auxin signaling, panicle development, and grain yield, requires precise modulation to optimize trade‐offs between spikelet number and grain quality.

To investigate natural variations in the *OsMADS47* gene, we analyzed coding sequences corresponding to the four phosphorylation sites using cultivars from the 3000 Rice Genomes Project (3KRG),^[^ ^60^ ^]^ and found these sites to be highly conserved. These four phosphorylation sites are also highly conserved even across different plant species (Figure S14, Supporting Information). By contrast, we detected nucleotide variations in the promoter region (Figure S17A, Supporting Information). Based on these promoter polymorphisms, we identified eight major haplotypes after filtering out rare variants (< 30 accessions) (Figure S17A, Supporting Information). Among them, Hap1 had the largest population size and was predominantly found in temperate geng (GJ‐tmp), while Hap2, Hap3, and Hap4 were mainly distributed in tropical geng (GJ‐trp), Xian (XI‐1A, XI‐1B, XI‐2, XI‐3 and XI‐adm), and subtropical geng (GJ‐sbtrp), respectively (Figure S17B, Supporting Information). Compared with Hap3 and Hap4, Hap2 exhibited a reduced length‐to‐width ratio, but its grain width showed a less pronounced decrease (Figure S17C, Supporting Information). This suggests that Hap2 distributed in tropical geng (GJ‐trp) may optimize grain shape while preserving yield potential, highlighting its value as an elite haplotype for breeding. Due to the limitations in the large‐scale cultivation of transgenic materials, we plan to perform backcross breeding using the elite haplotypes identified in this study and conduct field trials using non‐transgenic materials. We also aim to generate quantitative variations in *OsMADS47* expression levels through the CRISPR/Cas9‐mediated promoter editing technique in the future. Additionally, since the phosphorylation status of OsMADS47 is critical for its stability and activity, and no natural variations are observed at these sites, precise editing of the four phosphorylation sites identified in this study could further enhance its potential to improve rice grain yield and quality.

## 4 Conclusion

Our study identifies OsMADS47 as a critical transcription factor integrating G‐protein signaling, MAPK cascade, and BR signaling pathways. This integrative role provides a systematic framework for understanding the molecular and genetic mechanisms underlying grain shape regulation. Furthermore, our findings suggest a potential strategy to simultaneously improve grain yield and appearance quality through precise coordination of these pathways.

## 5 Experimental Section

### Plant Materials and Growth Conditions

Thirty‐four OsMADS‐box genes highly expressed in young panicles were subcloned into the *pCAMBIA1305‐APHA* vector in which the MADS‐box gene was fused with the HA‐tag and driven by the rice *Actin1* promoter and transformed into a *japonica* rice variety Nipponbare (NIP). The T_0_ transgenic plants were used for screening MADS‐box genes which regulate grain morphology. Overexpression of *OsMADS47* resulted in slender grains. Thus, *OsMADS47* was selected for further analysis and cloned into the *pCAMBIA1305* vector with the maize *Ubiquitin1* promoter to drive its expression. Stable transgenic lines (T_2_ generation) of *proActin:OsMADS47* and *proUbi:OsMADS47* were used for gene expression and phenotype analysis. All the plants were grown at two experimental stations of Chinese Academy of Agricultural Sciences located in Beijing and Hainan province, respectively, under natural growth conditions. Mature seeds were harvested and measured using a Seed Counter‐G system (Wseen, Hangzhou, China). For seedling analysis, plants were grown on half‐strength Murashige and Skoog medium in a growth chamber at 30 °C and 60% relative humidity in a 12‐h light/12‐h dark cycle.

### Vector Construction and Plant Transformation

A 1.8‐kb DNA fragment upstream of the *OsMADS47* start codon was amplified from NIP genomic DNA and cloned into the binary vector *pCAMBIA1305* to generate *proOsMADS47:GUS* vector. *OsMADS47* knockout plants were created by CRISPR/Cas9 technology. In brief, the target sequence (5′‐GGCACGGAGGATCGACAACCTGG‐3′) was synthesized, ligated with the intermediate sgRNA vector with *U3* promoter and cloned into the *pYLCRISPR/Cas9* binary vector. The full‐length coding sequence (CDS) of *GS3*, *GW8*, *PPKL1*, and *PPKL3* from NIP cDNAs was amplified and inserted into the *p2300‐35S‐GFP* binary vector to obtain *p35S:GS3‐GFP*, *p35S:GW8‐GFP*, *p35S:PPKL1‐GFP*, and *p35S:PPKL3‐GFP* constructs, respectively. A 200‐bp cDNA fragment was amplified from NIP cDNAs and inserted into the *pROKII‐RNAi* vector to generate *OsMPK6* RNAi construct. All the constructs were introduced into *Agrobacterium tumefaciens* strain EHA105 and then transformed into the corresponding rice materials. Relevant primer sequences are listed in Table S1 (Supporting Information).

### Subcellular Localization and Transcriptional Activity Analysis

To study subcellular localization of OsMADS47, the *OsMADS47* CDS was amplified and cloned into the *pAN580‐35S‐GFP* vector to obtain *p35S:OsMADS47‐GFP* construct. *D53* fused with *mCherry* was used as a nuclear localization marker as previously described.^[^ ^23^ ^]^ Both *p35S:OsMADS47‐GFP* and *p35S:D53‐mCherry* were cotransformed into the rice protoplasts and observed using a confocal laser scanning microscope (LSM710, Carl Zeiss, Jena, Germany).

To test the transcriptional activity of OsMADS47 in rice protoplasts, full‐length CDS of *OsMADS47* was amplified and cloned into the *GAL4BD* vector driven by the CaMV35S promoter. Four copies of the transcriptional repression domain EAR or transcription activation domain VP16 were fused with the OsMADS47 protein to generate GAL4BD‐OsMADS47‐EAR and GAL4BD‐OsMADS47‐VP16, respectively. Pro35S‐GAL4UAS‐LUC was adopted as the reporter vector as described previously.^[^ ^61^ ^]^ The *Renilla luciferase* (*REN)* gene driven by the CaMV35S promoter (35S‐RLUC) was used as an internal control. All the construct combinations were transiently transformed to the rice protoplasts with four independent replicates. Both the firefly luciferase (LUC) and REN activity were measured using a DLR assay kit (Promega, Madison, WI, USA) on a Centro XS3 LB 960 High Sensitivity Microplate Luminometer (Berthold Technologies, Bad Wildbad, Germany). To calculate the relative LUC activity (LUC/REN), the LUC activity was normalized to that of REN.

### In Situ Hybridization

*OsMADS47*‐specific probe was generated by inserting the cDNA fragment into *pMD18‐T* (TaKaRa; gene‐specific primers in Table S1, Supporting Information). RNA hybridization and immunological detection of the hybridized probes were performed as described previously.^[^ ^61^ ^]^

### DLR Assay

To generate reporter constructs, ≈3.0‐kb and 2.6‐kb promoter regions of *GS3* and *GW8* were amplified (Primer pairs listed in Table S1, Supporting Information) and inserted upstream of the firefly *LUC* gene in the pGreenII 0800‐LUC vector, respectively. The *REN* gene driven by CaMV35S promoter within this vector was used as an internal control. The full‐length CDS of *OsMADS47*, *OsMPK6*, and *PPKL1/3* were amplified and recombined into pGreenII 62‐SK vectors under the control of CaMV35S promoter. The resultant constructs were used as effector constructs. To test the direct effect of OsMADS47 on the expression of *GS3* and *GW8*, the construct combination of effector and reporter was co‐transformed into rice protoplasts as described in “Subcellular localization and transcriptional activity analysis”. To check the combined effects of OsMADS47, OsMPK6, and PPKL1/3 on the expression of *GS3* and *GW8*, the effector and reporter plasmid were transformed into *Agrobacterium tumefaciens* strain GV3101 (pSoup‐p19) and co‐infiltrated into 3‐week‐old *N. benthamiana* leaves. The LUC and RLUC activity were measured as described in “Subcellular localization and transcriptional activity analysis”.

### Luciferase Complementation Imaging Assay

Full‐length CDS of *OsMADS47* was cloned into the *pCAMBIA1300‐35SNLUC* vector. The full‐length CDS of *OsMPK6*, *PPKL1/3*, and *OsMADS47* were inserted into the *pCAMBIA1300‐35SCLUC* vector. All constructs were transformed into *Agrobacterium tumefaciens* strain GV3101 (*pSoup‐p19*) and co‐infiltrated into 3‐week‐old *N. benthamiana* leaves. The treated leaves were sprayed with 2.5 mm luciferin and imaged with Tanon‐5200 chemiluminescence imaging system (Tanon Science & Technology, Shanghai, China) at 72 h after infiltration.

### EMSA Assay

The MBP‐OsMADS47 construct was generated by cloning full‐length CDS of *OsMADS47* into the *pMAL‐MBP* vector. The recombinant protein MBP‐OsMADS47 was expressed in *E.coli* and purified using the amylose resin (E8021V, New England Biolabs, Beverly, MA, USA). The probes identified in the *GS3* and *GW8* promoters containing the CArG box (Table S1, Supporting Information) were synthesized and labeled with biotin at the 3′‐end. EMSAs were performed as described previously.^[^ ^61^ ^]^

### ChIP Assay

The ChIP assay was conducted as described previously.^[^ ^61^ ^]^ Briefly, nuclei were extracted and purified from young panicles of 1–2 cm in length of *OsMADS47*‐*HA* overexpression line (*OX‐4*) and WT. The nuclei extracts were sonicated into 0.2‐ to 1.0‐ kb fragments and were immunoprecipitated with anti‐HA (ab9110, Abcam, Cambridge, MA, USA) and Rabbit IgG serum, respectively, which was further collected with protein A agarose/salmon sperm DNA (16‐157, Merck Millipore, Billerica, MA, USA). The enrichment of DNA regions was quantified by qPCR, calculated by the percent input method and then compared with IgG serum control. The qPCR primers are listed in Table S1 (Supporting Information). The *Ubiquitin* gene and negative probes‐located regions were used as negative controls.

### Semi‐In Vivo Pull‐Down Assay

Full‐length CDS of *OsMPK6*, *PPKL1*, and *PPKL3* were cloned into the *pColdI‐GST* vector. The recombinant proteins were expressed in *E.coli* (*Transetta*). Shoots of HA‐OsMADS47 overexpression (*MADS47^ox^*) plants grown in 1/2 MS medium for 7 days were collected and total proteins were extracted using IP lysis buffer (50 mm Tris‐HCl pH 7.5, 1% Triton X‐100, 150 mm NaCl, 10% glycerol, 1 mm EDTA, 2.5 mm DTT, 50 µm MG132, and protease inhibitor cocktail). An equal amount of GST‐MPK6, PPKL1, or PPKL3 pre‐immobilized on GST beads was added and incubated at 4 °C for 1.5 h, respectively. Proteins retained on the beads were resolved by SDS–PAGE and detected with anti‐HA (HRP) (M20021, Abmart, Shanghai, China) and anti‐GST (HT601, TransGen Biotech, Beijing, China).

### Cell‐Free Protein Degradation Assay

Total proteins were extracted from 7‐day‐old NIP seedlings using extraction buffer (50 mm Tris‐HCl pH 7.4, 150 mm NaCl, 1% (v/v) NP‐40, and protease inhibitor cocktail). Equal amount of total protein extracts was incubated with equal amount of recombinant MBP‐OsMADS47, MBP‐OsMADS47 phosphorylated by OsMPK6, MBP‐OsMADS47 (Asp), MBP‐OsMADS47 (Ala) in a reaction system containing 25 mm Tris‐HCl (pH 7.5), 100 mm NaCl, 10 mm MgCl_2_, 5 mm DTT, 10 mm ATP, 0.5 µg recombinant proteins with 50 µm MG132 or DMSO for the indicated time at 30 °C. MBP‐OsMADS47 (Ala) and MBP‐OsMADS47 (Asp) was generated by introducing mutations at Thr‐70, Ser‐78, Thr‐79, Ser‐80 to aspartic acid and alanine, respectively. The proteins were collected and subjected to western blotting with anti‐MBP antibody. Actin was used as the loading control.

### In Vitro Kinase and Phosphatase Assays

The full‐length CDS of *OsMPK6* and *OsMKK4^CA^* harboring the Thr238Asp and Ser244Asp mutations were inserted into the pET28a vector, and the CDS of PPKL1/3 were cloned into the pCold‐GST vector. The recombined proteins MBP‐OsMADS47, His‐OsMPK6, His‐MKK4^CA^, and GST‐PPKL1/3 were expressed in *E.coli* and purified for kinase and phosphatase assays in vitro. For the kinase assay, His‐OsMPK6 was first incubated with His‐MKK4^CA^ at 2:1 ratio in kinase buffer (20 mm Tris‐HCl pH 7.5, 10 mm MgCl_2_, 100 mm NaCl, 2 mm dithiothreitol, 2 mm ATP) at 25 °C for 1 h. The reaction mixture containing activated His‐OsMPK6 was then used to phosphorylate MBP‐OsMADS47 (with a molar ratio of His‐OsMPK6:MBP‐OsMADS47 = 1:3) in the same kinase buffer at 30 °C for 2 h. For the phosphatase assay, the resultant kinase mixture was desalted with desalting columns (89882, Thermo Scientific, Waltham, MA, USA) to remove ATP and incubated with recombinant proteins GST‐PPKL1/3 (GST‐PPKL1/3:MBP‐OsMADS47 = 1:3) at 30 °C for 3 h in a reaction volume of 20 µL containing 50 mm Tris‐HCl pH 7.5, 10 mm MgCl_2,_ 10 mm MnCl_2_, 0.1 mm EDTA, 2 mm DTT and 0.01% Brij 35. All the samples in kinase and phosphatase assays were separated on 10% SDS‐PAGE gel with 40 µm Phos‐tag and 80 µm MnCl_2,_ followed by immunoblotting with anti‐MBP (M20051, Abmart, Shanghai, China), anti‐GST (HRP) (M20025, Abmart, Shanghai, China) and anti‐His (HT501‐01, TransGen Biotech, Beijing, China) antibodies.

### Identification of Phosphorylation Sites in *OsMADS47*

Young panicles of 1–2 cm in length from the *OsMADS47* expression plants (*OX‐4*) were sampled to isolate total proteins. The extracted proteins were incubated with anti‐HA agarose (KTSM1335, KT HEALTH, Shenzhen, China). The immunoprecipitated proteins were separated on SDS‐PAGE gel containing phos‐tag, and the target bands were subjected to liquid chromatography/tandem mass spectrometry (LC–MS/MS) assay.

### Statistical Analysis

All experimental data were analyzed and visualized using GraphPad Prism 9 software (GraphPad Software, San Diego, CA, USA). To evaluate statistical significance between two groups, the normality of the data distribution was first rigorously assessed using four established normality tests available in the software: the D'Agostino‐Pearson omnibus test, Anderson‐Darling test, Shapiro‐Wilk test, and Kolmogorov‐Smirnov test. Non‐parametric statistical methods were employed if any of these tests indicated non‐normality. Parametric tests were only applied when all four tests confirmed a Gaussian distribution, and these analyses were performed without assuming equal variances (heteroscedasticity). For comparisons involving three or more groups, one‐way ANOVA followed by post hoc multiple comparisons testing was used to determine significance. Results are presented as mean ± standard deviation (SD). Statistical significance was determined using a threshold of *p* < 0.05, with asterisks in figures indicating varying significance levels: * (*p* < 0.05), ** (*p* < 0.01), and *** (*p* < 0.001).

## Conflict of Interest

The authors declare no conflict of interest.

## Author Contributions

J.F. and Y.C. contributed equally to this work. X.L., Y.L., and J.F. conceived and designed the experiments. J.F., Y.C., F.Z., T.G., M.R., J.Z., S.Y., and W.W. performed the experiments. J.F., Y.L., and X.L. wrote and edited the article.

## Supporting information



Supporting Information

## Data Availability

All data supporting the findings of this study are available within the article and its Supporting Information. The primers used in this study are provided as Supplementary Table 1.
