# Supplementary material for: A Novel OsMPK6‐OsMADS47‐PPKL1/3 Module Controls Grain Shape and Yield in Rice
Source: Adv Sci (Weinh). 2025 Jun 5;12(30):e01946. doi: 10.1002/advs.202501946 (PMC12376620; doi:10.1002/advs.202501946)
Supplement: Supplementary file 1 — Supporting Information [file ADVS-12-e01946-s001.docx]

**Supporting Information**

**A novel OsMPK6-OsMADS47-PPKL1/3 module controls grain shape and yield in rice**

Jingjing Fang^1†^, Yan Chun^1†^, Fan Zhang^1^, Tingting Guo^2^, Mengmeng Ren^1^, Jinfeng Zhao^1^, Shoujiang Yuan^3^, Wensheng Wang^1^, Yunhai Li^4^ and Xueyong Li^1^*

1 State Key Laboratory of Crop Gene Resources and Breeding, National Key Facility for Crop Gene Resources and Genetic Improvement, Institute of Crop Sciences, Chinese Academy of Agricultural Sciences, Beijing 100081, China.

2 College of Agriculture and Biotechnology, Hunan University of Humanities, Science and Technology, Loudi 417000, China.

3 Institute of Wetland Agriculture and Ecology, Shandong Academy of Agricultural Sciences, Jinan 250100, China

4 State Key Laboratory of Plant Cell and Chromosome Engineering, CAS Centre for Excellence in Molecular Plant Biology, Institute of Genetics and Developmental Biology, The Innovative Academy of Seed Design, Chinese Academy of Sciences, Beijing 100101, China

†Jingjing Fang and Yan Chun contributed equally to this work.

*Correspondence and requests for materials should be addressed to X.L. (email: lixueyong@caas.cn)

**
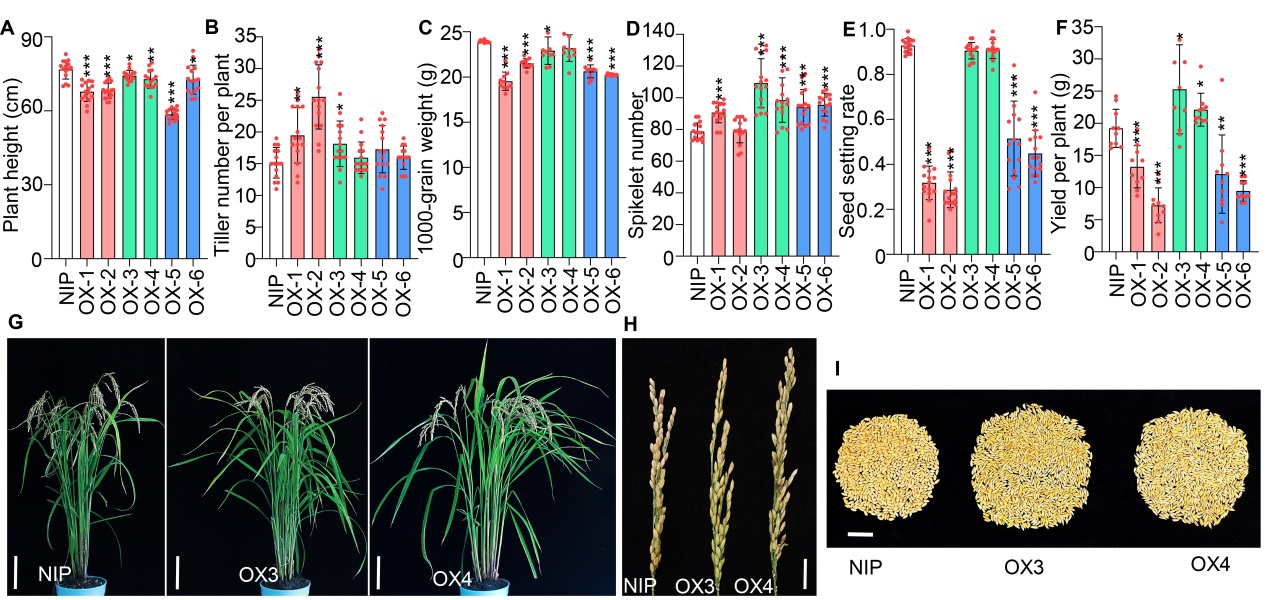
**

**Figure S1.** Phenotypic characterization of NIP and *OsMADS47* overexpression plants driven by the rice *actin1* promoter. A-F) Plant height (A), tiller number per plant (B), 1000-grain weight (C), spikelet number (D), seed setting rate (E) and yield per plant (F) in NIP and *OsMADS47*-overexpression lines (*n* = 15 in A, B, D, and E; *n* = 10 in C and F). Data are given as means ± SD. Student’s *t*-test was used to generate the *P* values; **P* < 0.05, ***P* < 0.01, ****P* < 0.001. G-I) Gross plant morphology (G), panicles (H) and grain yield per plant (I) of the moderate overexpression lines of *OsMADS47* (*OX3* and *OX4*). Scale bars, 10 cm (G), 5 cm (H) and 2.5 cm (I).


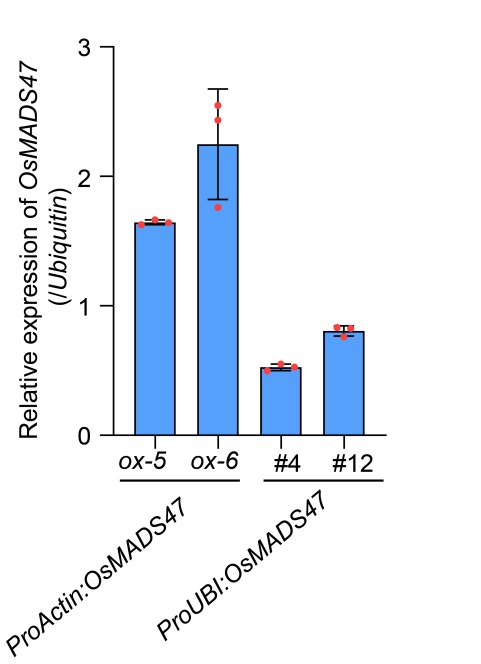


**Figure S2.** The relative expression of *OsMADS47* in OsMADS47 overexpression plants driven by rice *Actin* (*ProActin:OsMADS47*) and maize *Ubiquitin1* (*ProUBI:OsMADS47*) promoters. The *Ubiquitin* gene was used as an internal control (*n* = 3). Data are given as means ± SD.

**
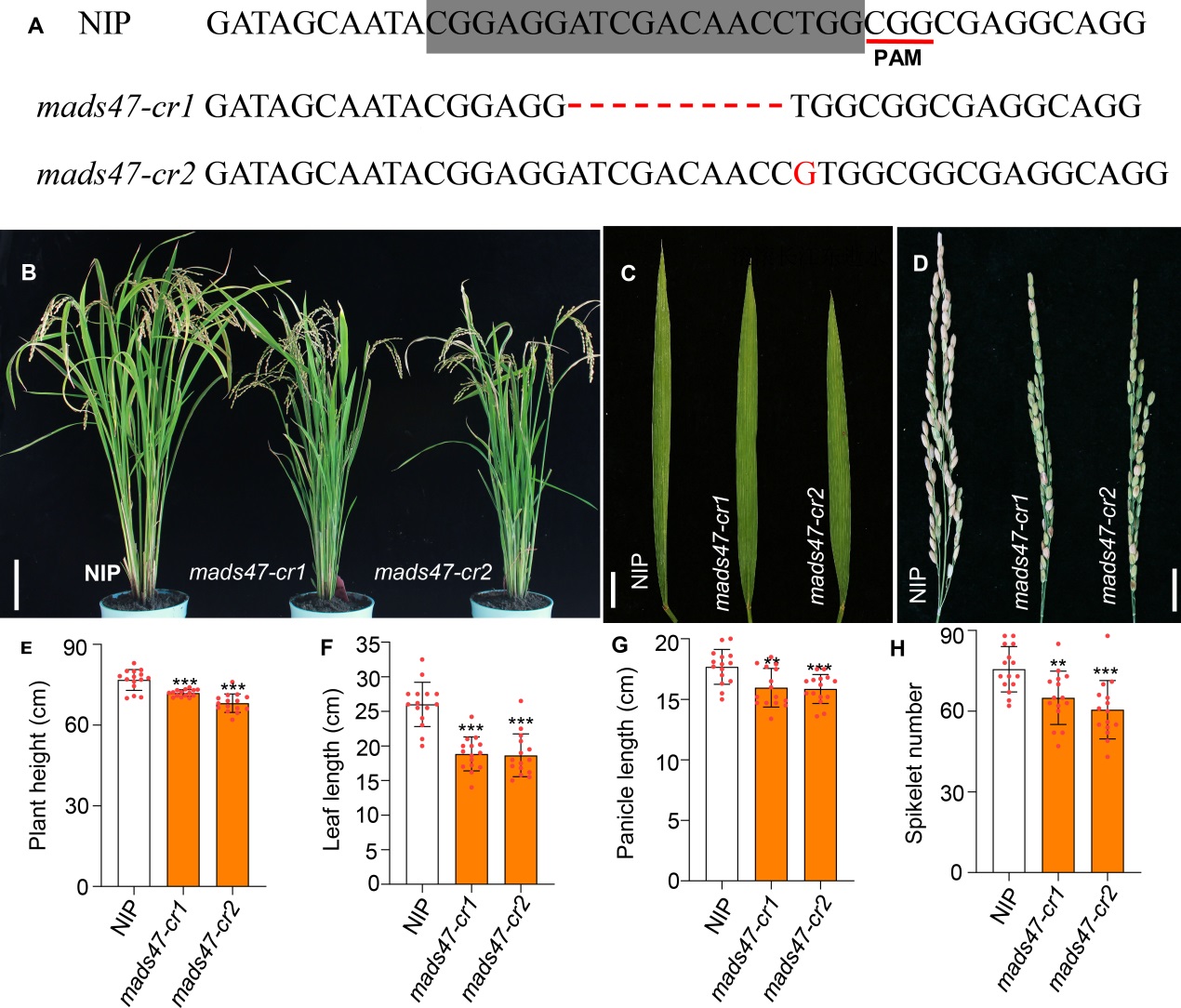
**

**Figure S3.** Phenotypic characterization of *OsMADS47* knockout lines. A) The mutation sites in the *OsMADS47* gene in the knockout lines (*mads47-cr1* and *mads47-cr2*) generated by CRISPR/Cas9. The sgRNA target sequence of NIP (wild type) is shaded in gray. Protospacer adjacent motif (PAM) sequence is underlined in red. Red dashes and letters indicate the number of nucleotides deleted and inserted at the target sequence, respectively. B) The gross morphology of *mads47-cr1* and *mads47-cr2* plants. Scale bar, 10 cm. C) Leaf morphology of NIP, *mads47-cr1* and *mads47-cr2* plants. Scale bar, 2.5 cm. D) Panicle morphology of NIP, *mads47-cr1* and *mads47-cr2* plants. Scale bar, 2 cm. E-H) Statistical analysis of plant height (E), leaf length (F), panicle length (G) and spikelet number (H) in NIP, *mads47-cr1* and *mads47-cr2* plants (*n* =15). Data are given as means ± SD. Student’s *t*-test was used to generate the *P* values; ***P* < 0.01, ****P* < 0.001.


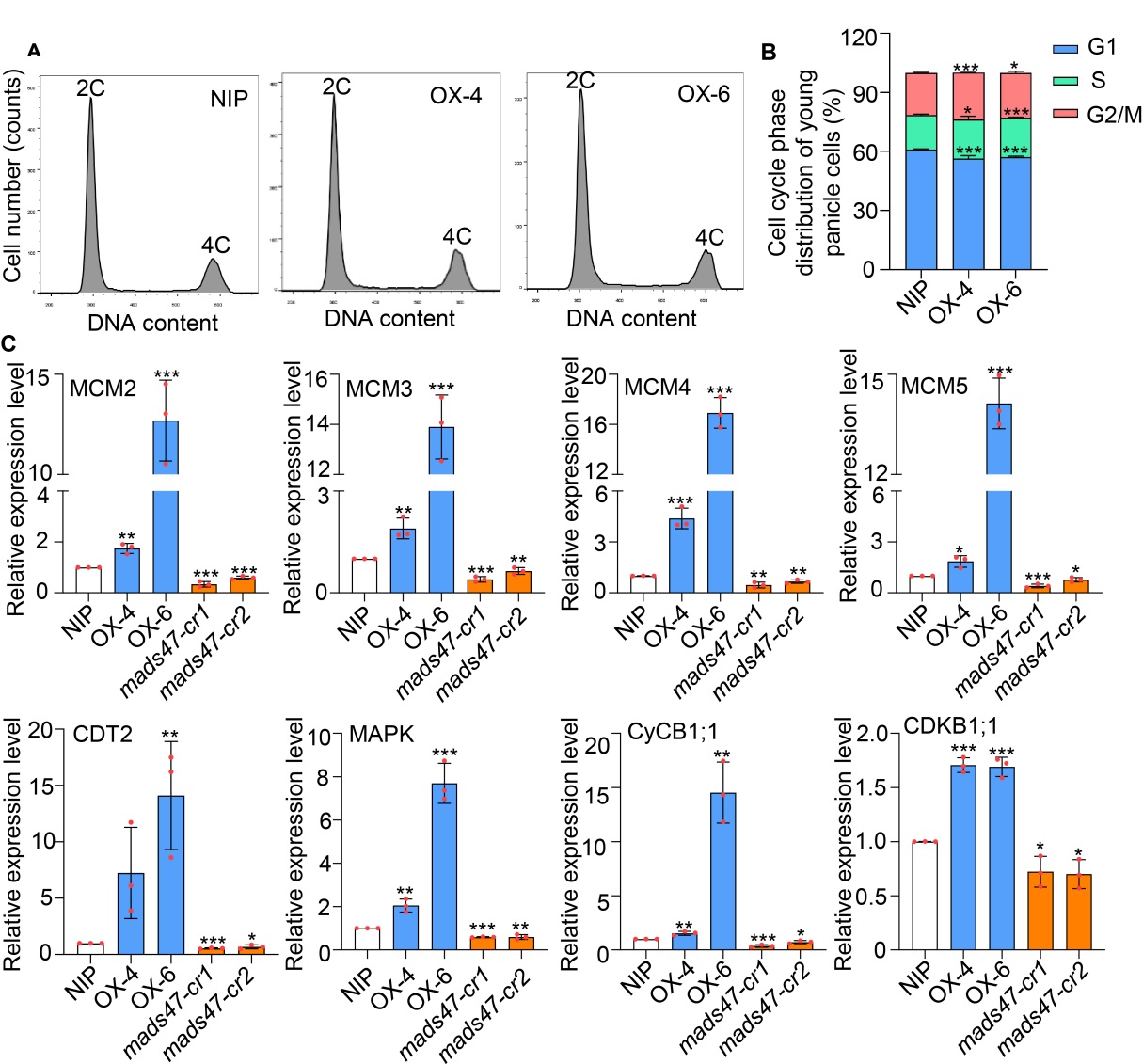


**Figure S4.** OsMADS47 regulates cell division in rice panicle. A) The cell number (counts) containing 2C DNA and 4C DNA in young panicles (1- to 2-cm long) of NIP and OsMADS47 overexpression lines (*OX-4* and *OX-6*) by flow cytometry analysis. B) Comparison of distribution percentage in different phases of cell cycle in young panicles of NIP, *OX-4* and *OX-6*. The percentage of cells in G1, S and G2/M phases are shown in colored boxes (*n* = 4). C) Analysis of relative expression level of cell cycle-related genes in young panicles (1- to 2-cm long) of NIP and *OsMADS47* transgenic plants (*OsMADS47* overexpression lines *OX-4* and *OX-6*; *OsMADS47* knockout lines *mads47-cr1* and *mads47-cr2*). *Ubiquitin* gene was used as an internal control (*n* = 3). In B and C, data are given as means ± SD. Student’s *t*-test was used to generate the *P* values; **P* < 0.05, ***P* < 0.01, ****P* < 0.001.


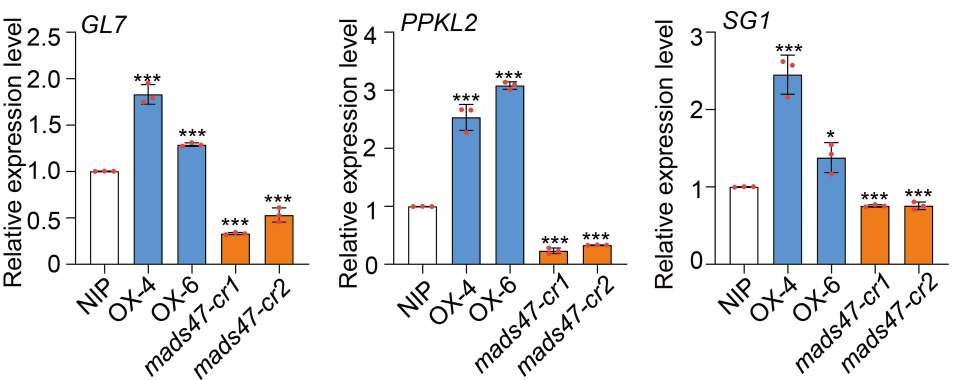


**Figure S5.** The relative expression of genes involved in the grain shape regulation. RNA isolated from young panicles of NIP and *OsMADS47* transgenic plants (*OX-4*, *OX-6*, *mads47-cr1* and *mads47-cr2*) was used for RT-qPCR analysis. *Ubiquitin* gene was used as an internal control (*n* = 3). Data are given as means ± SD. Student’s *t*-test was used to generate the *P* values; **P* < 0.05, ***P* < 0.01, ****P* < 0.001.


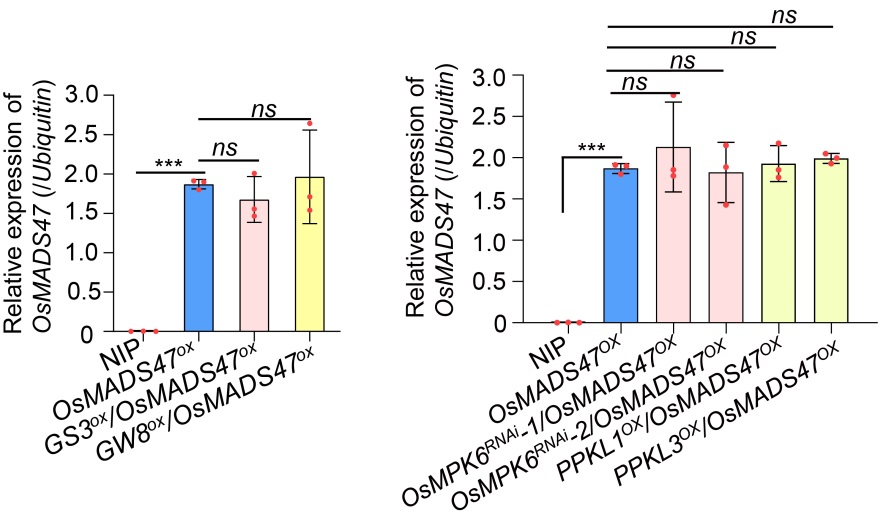


**Figure S6. The relative expression of *OsMADS47* in transgenic plants.** Relative expression of *OsMADS47* in NIP, *OsMADS47^OX^, GS3^OX^/OsMADS47^OX^*, *GW8^OX^/OsMADS47^OX^*, *OsMPK6RNAi/OsMADS47^OX^*, *PPKL1^OX^*/*OsMADS47^OX^* and *PPKL3^OX^*/*OsMADS47^OX^* plants. The *Ubiquitin* gene was used as an internal control (*n* = 3). Data are given as means ± SD**.** Student’s *t*-test was used to generate the *P* values; ****P* < 0.001, *ns* means non-significant (*P* > 0.05).

**
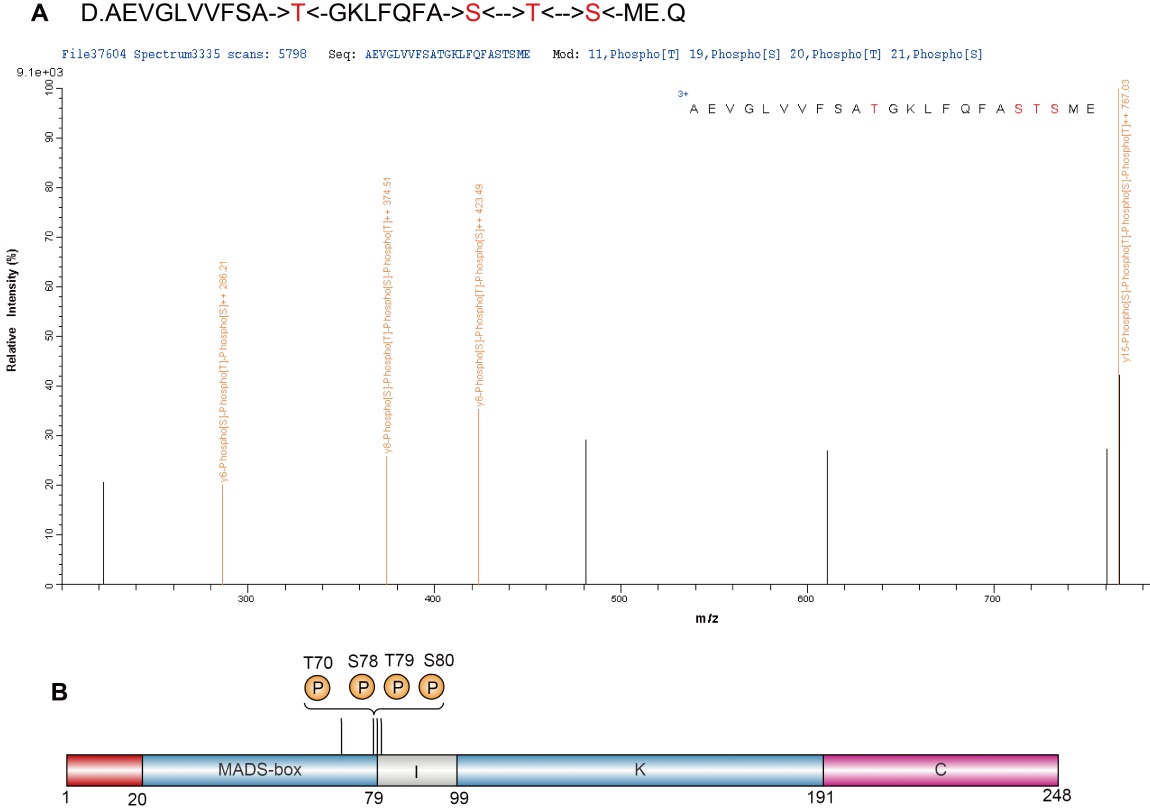
**

**Figure S7.** Identification of phosphorylation sites in OsMADS47 using LC-MS/MS. A) Potential phosphorylation sites of OsMADS47: T(70), S(78),T(79) and S(80). Young panicles of 1-2 cm in length from the *OsMADS47* overexpression plants (*OX-4*) were sampled to isolate total proteins. B) Locations of potential phosphorylation sites on each domains of OsMADS47. The numbering of amino acids corresponds to the OsMADS47 protein sequence. I, intervening domain; K, keratin-like domain; C, C-terminal domain.


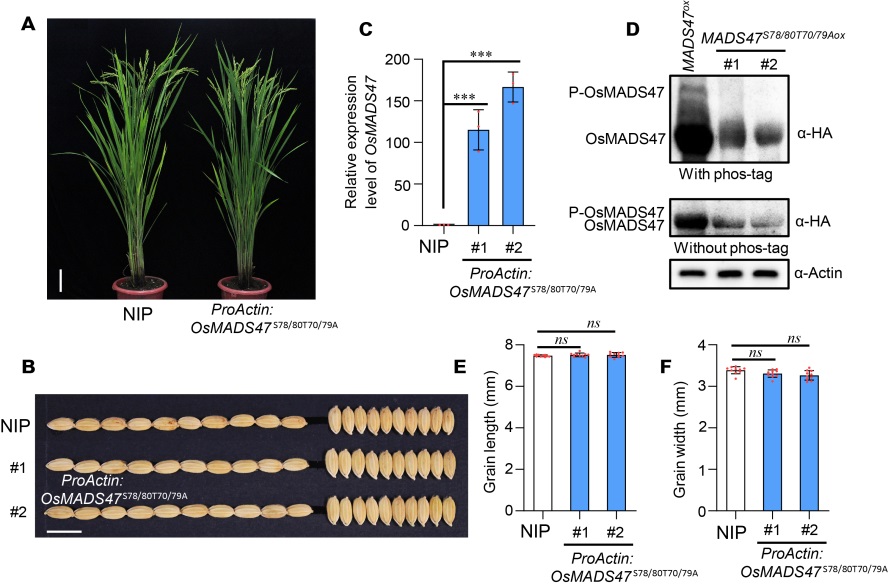


**Figure S8.** Phenotypic characterization of *ProActin:OsMADS47^S78/80T70/79A^* transgenic plants. A) The gross morphology of *ProActin:OsMADS47^S78/80T70/79A^* transgenic plants. Scale bar, 10 cm. B) Grain morphology of NIP and *ProActin:OsMADS47^S78/80T70/79A^* transgenic plants. Scale bar, 1 cm. C) Relative expression of *OsMADS47* in NIP and *ProActin:OsMADS47^S78/80T70/79A^* transgenic plants, normalized to the rice *Ubiquitin* gene (*n* = 3). D) The phosphorylated and total protein levels of in NIP and *ProActin:OsMADS47^S78/80T70/79A^* transgenic plants (*n* = 10). E,F) Grain length (E) and grain width (F) of NIP and *ProActin:OsMADS47^S78/80T70/79A^* transgenic plants (*n* = 10). Data are given as means ± SD. Student’s *t*-test was used to generate the *P* values; ****P* < 0.001; *ns*, no significant difference.


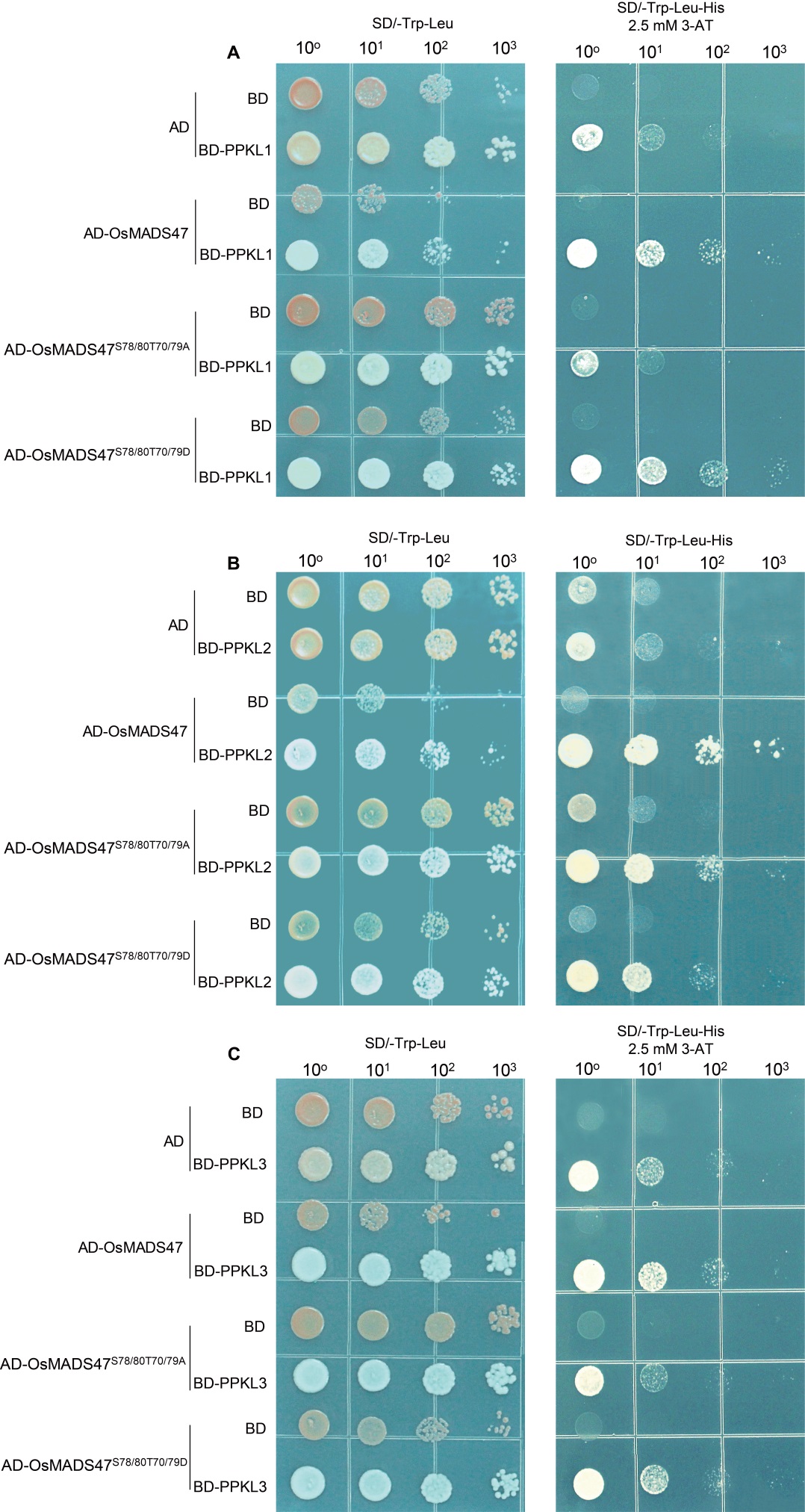


**Figure S9.** The phosphorylation status of OsMADS47 affects its interaction with PPKL1/2/3 in yeast cells. A-C) The interactions between OsMADS47, OsMADS47^S78/80T70/79A^ and OsMADS47^S78/80T70/79D^ with PPKL1 (A), PPKL2 (B) and PPKL3 (C), respectively. The AD-OsMADS47^S78/80T70/79A^ and AD- OsMADS47^S78/80T70/79D^ was generated by introducing mutations at Thr-70, Ser-78, Thr-79, Ser-80 to alanine and aspartic acid, respectively. The selection medium in (A) and (C) is SD/-Trp-Leu-His plus 2.5 mM 3-AT, while the selection medium in (B) is SD/-Trp-Leu-His.


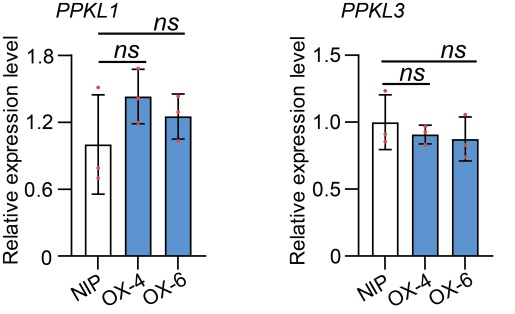


**Figure S10.** Relative expression of *PPKL1/3* in *OsMADS47* overexpression plants (*OX-4*, *OX-6*)*.* RNA isolated from young panicles of NIP, *OX-4* and *OX-6* was used for RT-qPCR analysis. *Ubiquitin* gene was used as an internal control (*n* = 3). Data are given as means ± SD. Student’s *t*-test was used to generate the *P* values; *ns*, no significant difference.


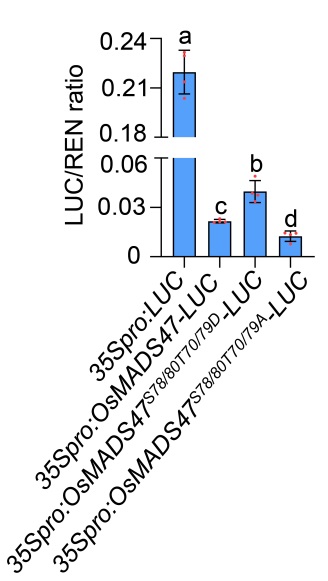


**Figure S11.** Phosphorylation status of OsMADS47 affects its stability in transient expression assay in rice protoplasts using the dual-luciferase system. Different letters indicate statistically significant differences at *P* < 0.01 determined by one-way ANOVA.

**
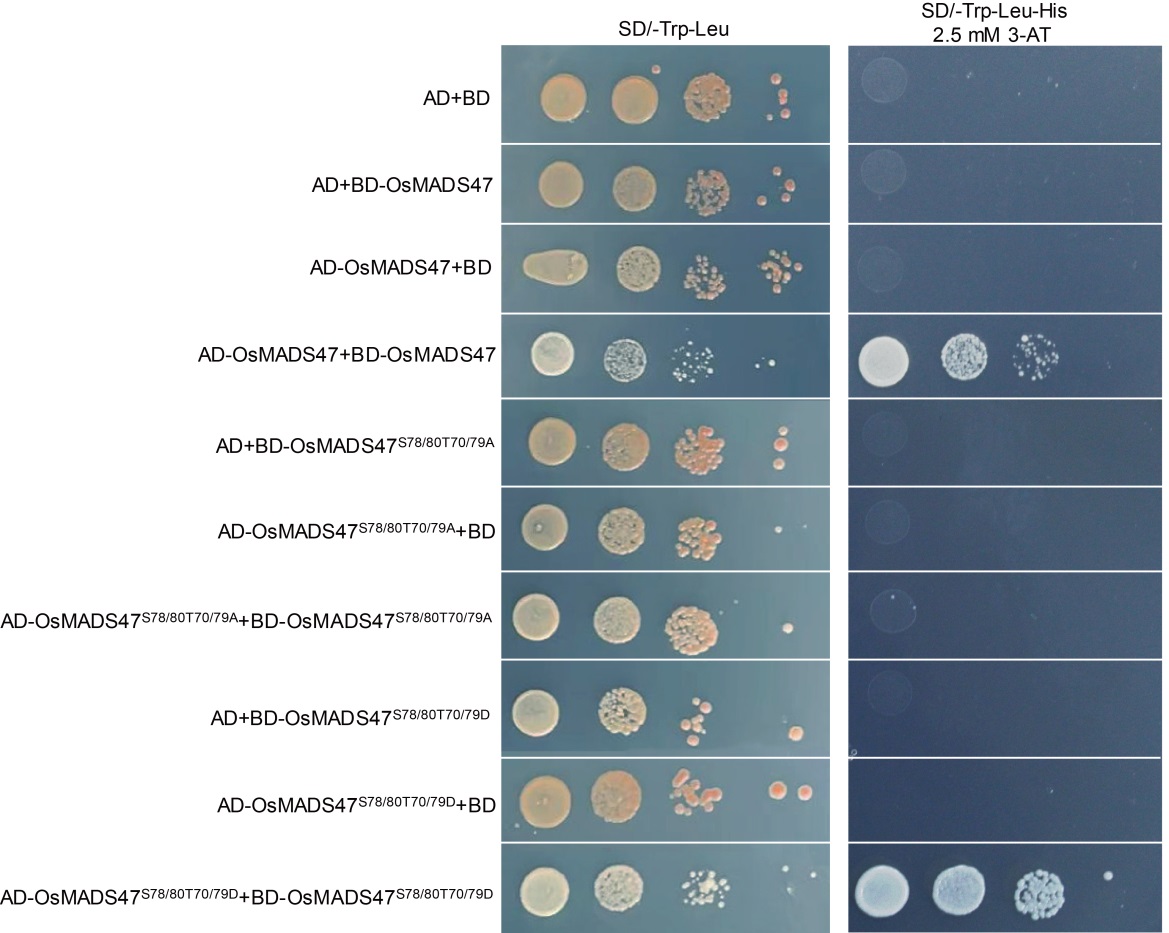
**

**Figure S12.** Homodimerization of OsMADS47, phosphorylation-mimicking OsMADS47^S78/80T70/79D^ and dephosphorylation-mimicking OsMADS47^S78/80T70/79A^ mutant protein. The full-length coding sequences of *OsMADS47*, *OsMADS47^S78/80T70/79D^* and *OsMADS47^S78/80T70/79A^* were cloned into pGADT7 (AD) and pGBKT7 (BD) vectors, respectively. The empty AD and BD vectors were used as negative controls. The indicated construct combinations were transformed into yeast cells and spotted onto control medium (SD/-Trp-Leu) and selective medium (SD/-Trp-Leu-His supplemented with 2.5 mM 3-AT).

**
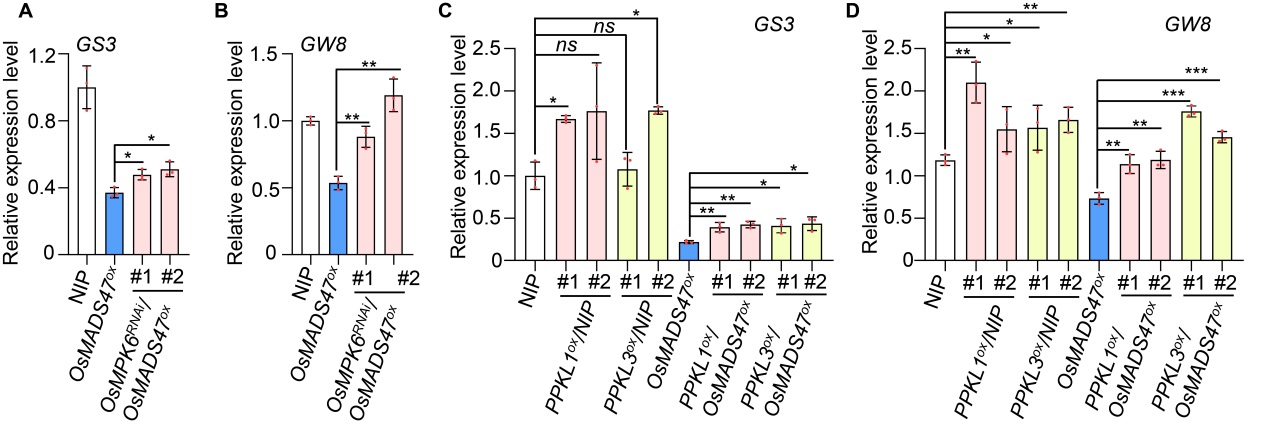
**

**Figure S13.** The *GS3* and *GW*8 expression levels in *OsMPK6^RNAi^*/*OsMADS47^OX^* and *PPKL1/3^OX^*/*OsMADS47^OX^* plants. A,B) The *GS3* (A) and *GW8* (B) expression levels in NIP, *OsMADS47^OX^* and *OsMPK6RNAi/OsMADS47^OX^* plants. C,D) The *GS3* (C) and *GW8* (D) expression levels in *PPKL1*-overexpression plants in NIP or *OsMADS47^OX^* background (*PPKL1^OX^*/NIP, *PPKL1**^OX^*/*OsMADS47^OX^*) and *PPKL3*-overexpression plants in NIP or *OsMADS47^OX^* background (*PPKL3^OX^*/NIP, *PPKL3^OX^*/*OsMADS47^OX^*). *Ubiquitin* gene was used as an internal control (*n* = 3). Data are given as means ± SD. Student’s *t-*test was used to generate the *P* values; **P* < 0.05, ***P* < 0.01, ****P* < 0.001; *ns*, no significant difference.


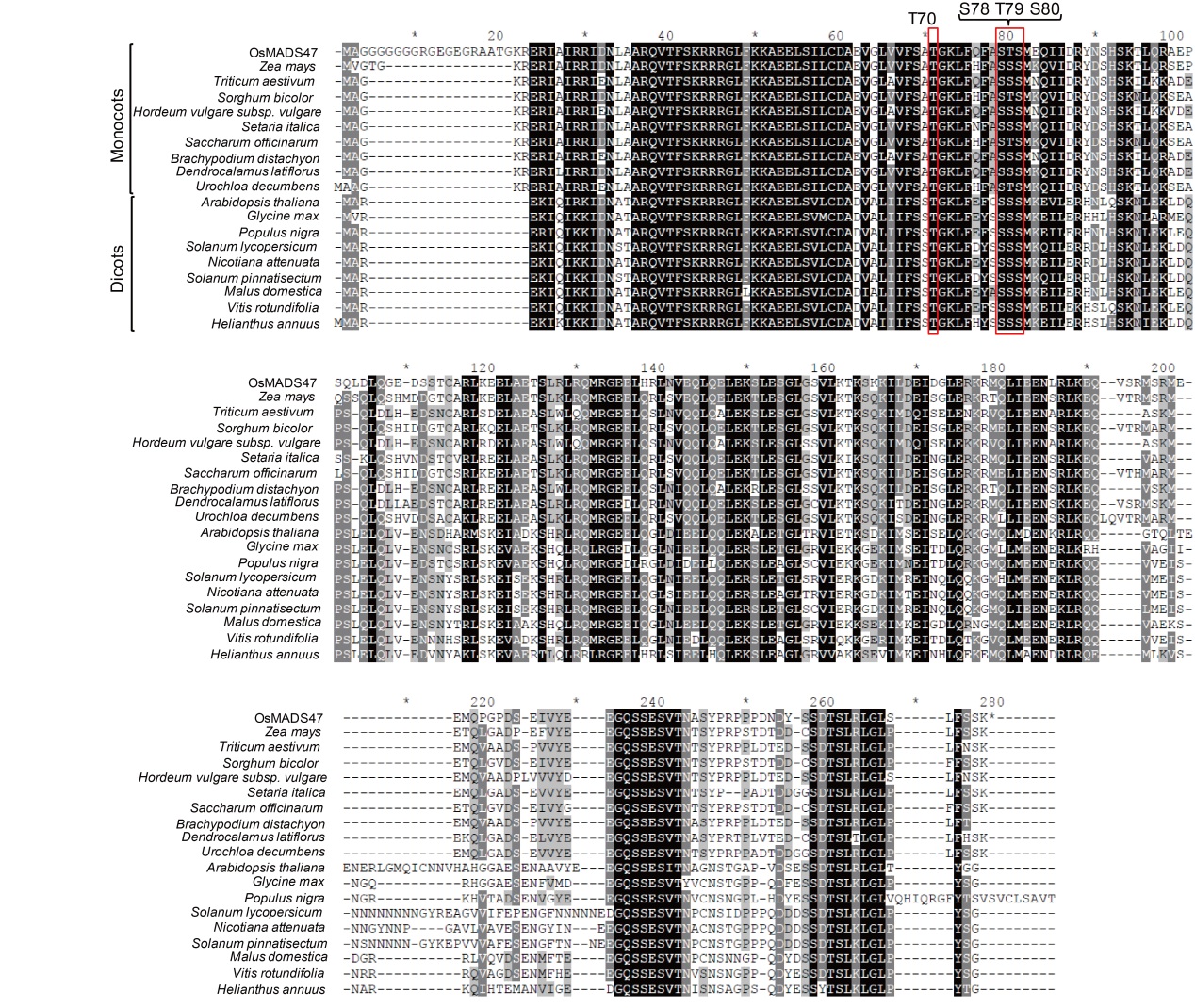


**Figure S14.** Protein sequence alignment of OsMADS47 and homologs from diverse plant species using the ClustalW analysis tool. Alignments are for NP_001306702.1 (*Zea mays*), XP_044376704.1 (*Triticum aestivum*), XP_021307342.1 (*Sorghum bicolor*), XP_044982876.1 (*Hordeum vulgare subsp. Vulgare*), XP_012698351.1 (*Setaria italic*), ACH86229.1 (*Saccharum officinarum*), NP_001288323.1 (*Brachypodium distachyon*), ASZ80017.1 (Dendrocalamus latiflorus), CAL4929468.1 (*Urochloa decumbens*), BAE98676.1 (*Arabidopsis thaliana*), NP_001240951.1 (*Glycine max*), XP_061950297.1 (*Populus nigra*), NP_001306770.1 (*Solanum lycopersicum*), XP_019261700.1 (*Nicotiana attenuate*), KAK4707750.1 (*Solanum pinnatisectum*), XP_028953815.1 (*Malus domestica*), KAJ9670234.1 (*Vitis rotundifolia*), XP_022001247.1 (*Helianthus annuus*). Dashes (–) indicate gaps introduced to maximize alignment. Identical amino acid residues are indicated by white-on-black lettering. The red boxes highlight the phosphorylation sites in OsMADS47 (Thr-70, Ser-78, Thr-79, and Ser-80) and their homologous sequences in other species.


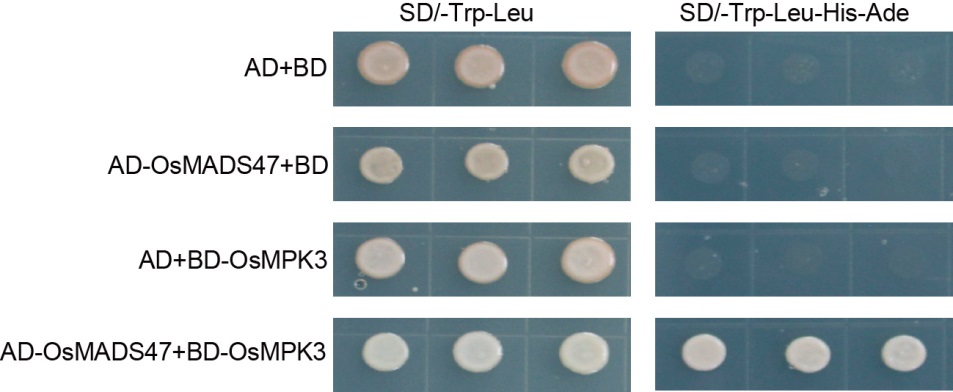


**Figure S15.** OsMPK3 physically interacts with OsMADS47 in yeast cells. The full-length coding sequence of *OsMADS47* and *OsMPK3* were cloned into pGADT7 (AD) and pGBKT7 (BD), respectively. The indicated construct pairs were co-transformed into yeast cells and spotted on control medium (SD/-Trp-Leu) and selective medium (SD/-Trp-Leu-His-Ade).


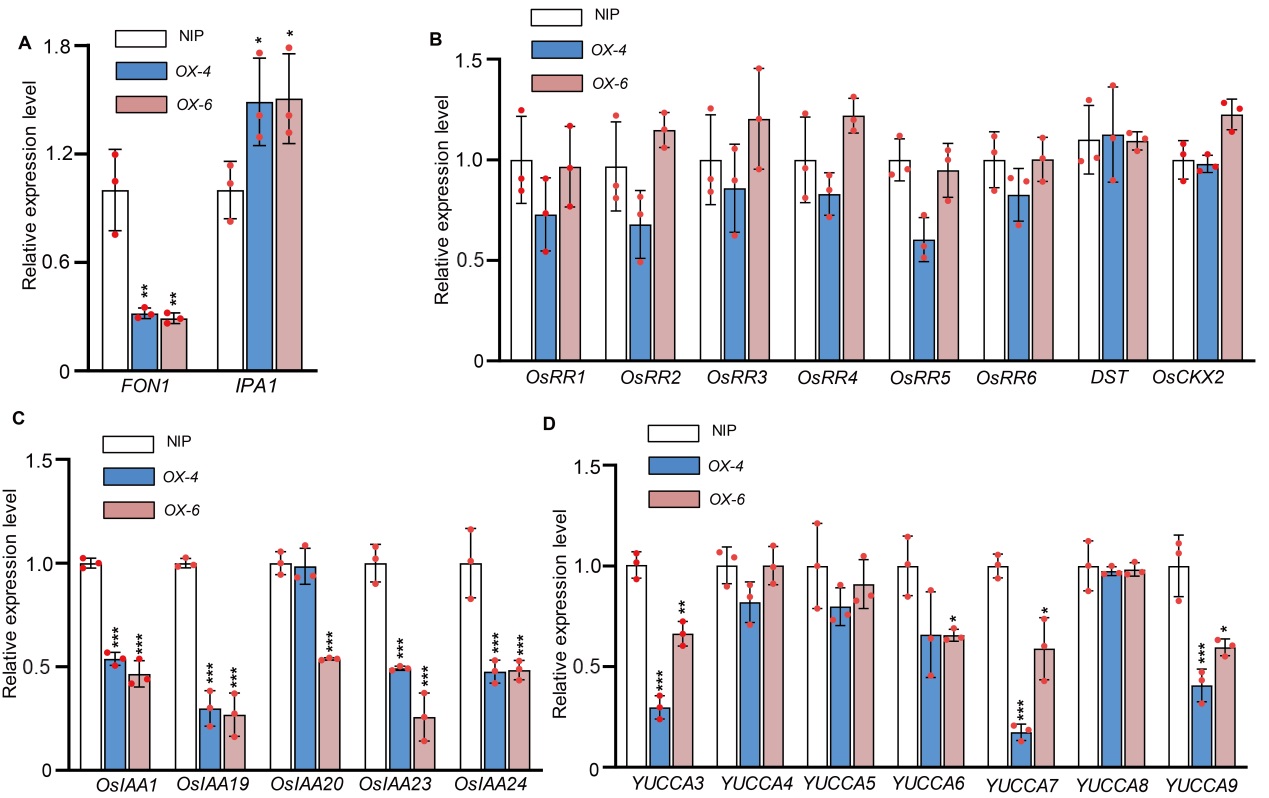


**Figure S16.** Expression of genes involved in panicle development in WT and *OsMADS47* overexpression plants. A-D) Analysis of relative expression level of two major panicle development regulators (*FON1* and *IPA1*) (A), cytokinin-related genes (Type-A Response Regulators, *DST* and *OsCKX2*) (B), and auxin-related genes (genes of the *Aux/IAA* and *YUCCA* families) (C,D) in NIP and *OsMADS47* overexpression plants (*OX-4* and *OX-6*). *Ubiquitin* gene was used as an internal control (*n* = 3). Data are given as means ± SD. Student’s *t*-test was used to generate the *P* values; **P* < 0.05, ***P* < 0.01, ****P* < 0.001.


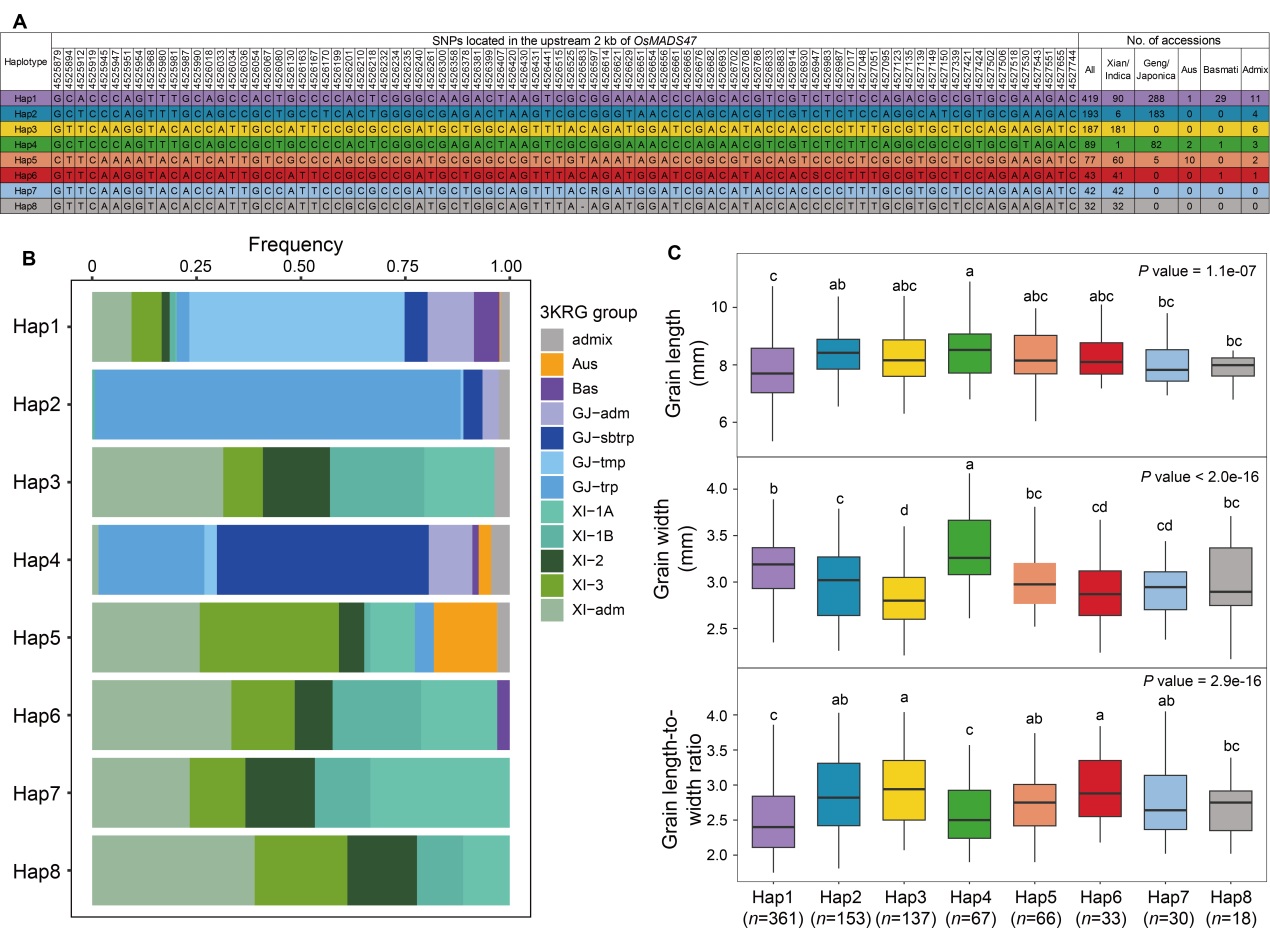


**Figure S17.** Haplotype analysis of the *OsMADS47* gene. A) DNA polymorphisms in the promoter region (2 Kb upstream) of *OsMADS47* in 3000 rice accessions; Positions of variations are indicated in the diagram. B) Haplotype frequency of *OsMADS47* in subpopulations from the 3,000 Rice Genomes Project (3KRG); admix, admixture; Aus, Aus; Bas, Basmati;GJ-adm, ; GJ-sbtrp, subtropical geng; GJ-tmp, temperate geng; GJ-trp, tropical geng; XI-1A, xian-1 A; XI-1B, xian-1 B; XI-2, xian-2; XI-3, xian-3; XI-adm, xian-admixture; C) Performance of different haplotypes of  *OsMADS47* on **grain length, grain width, and grain length-to-width ratio** in 3000 rice accessions**. Different lowercase letters on top of boxplots indicate significant differences** (Duncan’s test, *P* < 0.05)**.**

**Table S1.** Primers used in this study

|  |  | |  |
| --- | --- | --- | --- |
| **Vector construction for plant transformation** | | |  |
| OsMADS47 overexpression | | tatcgataccgtcgacatggctggcggcggcggtg | gtcaccaattcacacgtgtcacttggagctgaagagtg |
| OsMADS47 knock-out | | ggcacggaggatcgacaacctgg | aaacccaggttgtcgatcctccg |
| OsMPK6 knock-down | | cacgggggactctagaggatccatggacgccggggcgcagcc | ctagaaatttacccccgggttgtacttggcggtgacctc |
|  | | cgatcggggaaattcgagctcatggacgccggggcgcagcc | gttacagaactagtcaggtaccttgtacttggcggtgacctc |
| PPKL1 overexpression | | gcggcagcggccgaattcatggacgtggactcccgcatgac | cgcggccccggtggatccctatatccaggcaagagaac |
| PPKL3 overexpresssion | | gcggcagcggccgaattcatggacgtggactcgag | cgcggccccggtggatccctatatccatgcaagagagcctc |
| GS3 overexpression | | gcggcagcggccgaattcatggcaatggcggcggcgccccggcccaag | cgcggccccggtggatcctcacaagcagggggggcagcaac |
| GW8 overexpression | | gcggcagcggccgaattcatggagtgggatctcaagatg | cgcggccccggtggatccctactgccatgagaacggcagagac |
| **qRT-PCR** | |  |  |
| OsMADS47 | | caaggggaggacagcagtac | ctatcaattgcatcctctttcgttc |
| GS3 | | gaactcctgatccattcataacgatt | caaacagcgaaacttcttcaagaa |
| GW8 | | gaaagcgactagatgggcacaac | ggatttggtggtgcgtgtagtat |
| MPK6 | | aggtcaccgccaagtacaag | agcagcttgatctccctgag |
| PPKL1 | | taacaacatgacgcctccacctg | gtttctgcagccactacaacagc |
| PPKL3 | | tgaggacacatggatgcaggaac | tgcaagagagcctcggtcattg |
| MCM2 | | aagttggcaaaagatccacgg | cccccaaacatagctagtgcaa |
| MCM3 | | ttcatgcgtcactaaatgcgag | tgaatctggaagcccaatgttc |
| MCM4 | | cccgaatgcgattctctgaa | accagtggcatgatcagttgc |
| MCM5 | | aaggagaactgcctgtccatga | agtggccttagctttcaccctc |
| CDT2 | | aaccgcaccaaacactggaa | gcaattcaccatctgcactgg |
| MAPK | | acagagcagccgaattttgaga | ttcagcgaagctcacacttgg |
| CyCB1;1 | | cactctcaagcaccacactgga | acaaccctcagcttgctctcag |
| CDKB1;1 | | caccaacgtcatcaagagcttc | ccaagatcagcaattttcaatatcc |
| OsBRI1 | | cttggttcccttgccacattt | cctcattcttcagcctccgc |
| D11 | | gcagctaccccaggccaattc | gcaatcttctcaatgtcgccaag |
| LG3 | | ctgttccagctcccctactcc | caaactgcatcaatggaggagtac |
| GL7 | | ctgatggggttggaagaagcac | gttttcattgccggagactcatc |
| PPKL2 | | cgagatagcttctggtttcagcac | acgccatacagcactaacagc |
| SG1 | | cccagatggatcttggaggt | ggcagacagcaagctgaaag |
| OsCKX2 | | cagatttccaacgtgctagagctc | gtccggcgccttctccttc |
| OsRR1 | | cttcgctggagttgccat | tcaagcacaccacaggtt |
| OsRR2 | | gacatcgtgctgaccgact | atcgttcatcttgagaggctt |
| OsRR3 | | cggagatgacagggttcg | atttcatgatgacgcggttg |
| OsRR4 | | caccagataagccgccaca | tggaggacaatcttggctt |
| OsRR5 | | gggaagagggcattggag | cttcctccaagcacctgt |
| OsRR6 | | cgtcatcgccaagatcctc | cgggatctccttgagctg |
| DST | | caaggacgtgcggctgttc | cggcatgtagaagtagggattc |
| FON1 | | cttctgaaccccaaacttaacg | cagaaaagcagtagtaatccgc |
| IPA1 | | caagggttccaagcagcgtaa | tgcacctcatcaagtgagac |
| YUCCA3 | | tgggctaaagcgtcctacca | tgtgccgacgtcaagaactg |
| YUCCA4 | | aacggtggattggctggtact | cgtcttacccgacacactcttg |
| YUCCA5 | | gcctcgacctctgcaacaat | ccctaggcagaacatgaatcttg |
| YUCCA6 | | catactggccaccggatacc | acccctcttgtgtgaagaagtca |
| YUCCA7 | | cggctaccgcagcaatgt | gggtacccgtccttgttgaa |
| YUCCA8 | | gcaggtgggttgtgacaaagt | ttcagccgtatctgcattgc |
| YUCCA9 | | tcggcctagaaagaaattaaatcaa | tccacaaactcagaaacaaaatcatag |
| OsIAA1 | | ccagcgccttcgtctcat | gcggctcttggtgctaagtt |
| OsIAA19 | | tgccaaggcacaagttgttg | gccaatgtattcttccggtagct |
| OsIAA20 | | caccttatgttcccctccactaac | ggtgacagcgtatgggtgac |
| OsIAA23 | | aatctgatccgatcaatcaacac | cagaatcgaaatgtcattcactca |
| OsIAA24 | | gctgagggaggcactggat | tcagaggcggttgcagaga |
| **Subcellular localization** | |  |  |
| OsMADS47 | | ctcgaggaccggtcccgggatggctggcggcggcggtggc | cttgctcaccatggatcccttggagctgaagagtgac |
| **Transcriptional activity analysis** | | |  |
| OsMADS47-GAL4BD | tgtatcgccgagatctatggctggcggcggcggtggc | | tagactaggtggatcctcacttggagctgaagag |
| OsMADS47-VP16 | tgtatcgccgagatctatggctggcggcggcggt | | tccagcgcgtccatggtacccttggagctgaagagtgac |
| **In situ hybridization** |  | |  |
| OsMADS47 | cttgcatgcctgcagctcgggttgtcactcttcagctc | | ttcgagctcggtaccgtatgggagtagaattcaggtaacatcc |
| **Dual-luciferase reporter assay** | | |  |
| OsMPK6-NLUC | gctcgagtagtcgacatggacgccggggcgcagccgc | | cgagatctggtcgacctggtaatcagggttgaac |
| PPKL1-NLUC | gctcgagtagtcgacatggacgtggactcccgcatgac | | cgagatctggtcgactatccaggcaagagaac |
| PPKL3-NLUC | gctcgagtagtcgacatggacgtggactcgag | | cgagatctggtcgactatccatgcaagagagcctc |
| OsMADS47-NLUC | gctcgagtagtcgacatggctggcggcggcggtggc | | cgagatctggtcgaccttggagctgaagag |
| OsMADS47-CLUC | tcccggggcggtaccatggctggcggcggcggtggc | | gctctgcaggtcgactcacttggagctgaagag |
| **Yeast two-hybrid assay** |  | |  |
| AD-OsMADS47 | ggaggccagtgaattcatggctggcggcggcggtggc | | cgagctcgatggatcctcacttggagctgaagag |
| BD-OsMADS47 | gccatggaggccgaattcatggctggcggcggcggtggc | | ctgcaggtcgacggatcctcacttggagctgaagag |
| BD-OsMPK6 | gccatggaggccgaattcatggacgccggggcgcagccgc | | ctgcaggtcgacggatccctactggtaatcagggttgaac |
| BD-PPKL1 | gccatggaggccgaattcatggacgtggactcccgcatgac | | ctgcaggtcgacggatccctatatccaggcaagagaac |
| BD-PPKL3 | gccatggaggccgaattcatggacgtggactcgag | | ctgcaggtcgacggatccctatatccatgcaagagagcctc |
| BD-OsMPK3 | gccatggaggccgaattcatggacggggcgccggtg | | ctgcaggtcgacggatccctagtaccggatgtttgggttc |
| **EMSAs** |  | |  |
| GS3-P1 | gctgcacaacccgcgatgccgtcggccggatttgggaggtggagcgtcgccgccctcc | | ggagggcggcgacgctccacctcccaaatccggccgacggcatcgcgggttgtgcagc |
| GS3-P1 mutated probe | gctgcaccacccgcgatgccgtcggaggtggagcgtcgccgccctcc | | ggagggcggcgacgctccacctccgacggcatcgcgggtggtgcagc |
| GS3-P2 | ctctcccttccatcattacttgcccaaaaacggcaatcccctcccctccatctccatgtg | | cacatggagatggaggggaggggattgccgtttttgggcaagtaatgatggaagggagag |
| GS3-P2 mutated probe | ctctcccttccatcattacttgcaatcccctcccctccatctccatgtg | | cacatggagatggaggggaggggattgcaagtaatgatggaagggagag |
| GS3-P3 | gccaccctccctacgacgtagccagatatgggtggagaggtaagcgccgccgccgctgc | | gcagcggcggcggcgcttacctctccacccatatctggctacgtcgtagggagggtggc |
| GS3-P3 mutated probe | gccaccctccctacgacgtagcggagaggtccgcgccgccgccgctgc | | gcagcggcggcggcgcggacctctccgctacgtcgtagggagggtggc |
| GS3-P4 | ccaccgtccccccttccccctccaaccagatctgggaggaagggagggagggaggggag | | ctcccctccctccctcccttcctcccagatctggttggagggggaaggggggacggtgg |
| GS3-P4 mutated probe | ccaccgtccccccttccccctccagaggcagggagggagggaggggag | | ctcccctccctccctccctgcctctggagggggaaggggggacggtgg |
| GS3-P5 | cttttctaaacgatattcttcatgaaccaaaggagggaagctcattccgaaaatttttcc | | ggaaaaattttcggaatgagcttccctcctttggttcatgaagaatatcgtttagaaaag |
| GS3-P5 mutated probe | cttccctacacgaccctcttcatgaccagctcattccgaacccccttcc | | ggaagggggttcggaatgagctggtcatgaagagggtcgtgtagggaag |
| GW8-P1 | cttatagtccaacaatttgagagcaattaatggtcaaaaattaaatgaaaaatagaaattc | | gaatttctatttttcatttaatttttgaccattaattgctctcaaattgttggactataag |
| GW8-P1 mutated probe | cttatagtccaacaatttgagagtcaaaaattaaatgaaaaatagaaattc | | gaatttctatttttcatttaatttttgactctcaaattgttggactataag |
| GW8-P2 | gtaactgactacccccagttcctgccctcttgggccttggtattgttgctccaag | | cttggagcaacaataccaaggcccaagagggcaggaactgggggtagtcagttac |
| GW8-P2 mutated probe | gtaactgactacccccagttcctccttggtattgttgctccaag | | cttggagcaacaataccaaggaggaactgggggtagtcagttac |
| GW8-P3 | ctactttccttattccactagaaatcatatatacctttctcactctacataatc | | gattatgtagagtgagaaaggtatatatgatttctagtggaataaggaaagtag |
| GW8-P3 mutated probe | ctactttccttcctccactagaacccttctcactctacataatc | | gattatgtagagtgagaagggttctagtggaggaaggaaagtag |
| **ChIP assay** |  | |  |
| GS3-P1 | ccgctgcacaacccacgat | | cccatcgctctctttcctcagac |
| GS3-P2 | catccactccactctccactctctc | | gcggagctaggtggaagagc |
| GS3-P3 | gggagggcagggaaggttc | | catcgtgggttgtgcagc |
| GS3-P4 | catgtctgaggaaagagagcgatg | | gtgatggctcccctccctc |
| GS3-CK | ggctaaatccacccgtttgc | | cccaaaaccataaccctagatcc |
| GW8-P1 | ggtaaaacttttattcacttatagtccaac | | agtacttgtttactctcatttcttaataacc |
| GW8-P2 | gtttcttgcaagtactgagtaactgactac | | ggaaagtaggagtagtggcaaagatc |
| GW8-P3 | gatctttgccactactcctactttcc | | gagagggggattatgtagagtgag |
| GW8-CK | gctttaaaaacacaacctttcacctc | | gcatgctttgatctttttgcac |
| ***Semi*-in vivo pull-down assay** | | |  |
| MPK6 | catatggagctcggtaccatggacgccggggcgcagccgc | | gacaagcttgaattcctactggtaatcagggttgaac |
| **In vivo co-immunoprecipitation** | | |  |
| OsMADS47 | tatcgataccgtcgacatggctggcggcggcggtggc | | ttgcggagtacccgggtacctcacttggagctgaagag |
| OsMPK6 | tatcgataccgtcgacatggacgccggggcgcagccgc | | ttgcggagtacccgggtaccctactggtaatcagggttgaac |
| PPKL1 | tatcgataccgtcgacatggacgtggactcccgcatgac | | ttgcggagtacccgggtacctcaagctctgagctcacggatg |
| PPKL3 | tatcgataccgtcgacatggacgtggactcgag | | ttgcggagtacccgggtaccctatatccatgcaagagagcctc |
| **In vitro kinase and dephosphatase assays.** | | |  |
| OsMADS47 | \| ctcgagggatccgaattcatggctggcggcggcggtg \| ctgcaggtcgacaagctttcacttggagctgaagagtg \| \| --- \| --- \| | | \| ctgcaggtcgacaagctttcacttggagctgaagagtg \| ctgcaggtcgacaagctttcacttggagctgaagagtg \| \| --- \| --- \| |
| OsMPK6 | gtcgcggatccgaattcatggacgccggggcgcagccgc | | gtgcggccgcaagcttctactggtaatcagggttgaac |
| OsMKK4^CA^ | gtcgcggatccgaattcatgcgaccgggcgggccgccgagcttg | | gtgcggccgcaagctttcatgacggaggcggtgcgag |
| PPKL1 | catatggagctcggtaccatggacgtggactcccgcatgac | | gacaagcttgaattcctatatccaggcaagagaac |
| PPKL3 | catatggagctcggtaccatggacgtggactcgag | | gacaagcttgaattcctatatccatgcaagagagcctc |

**Experimental Section**

*Histological Analysis:* Analysis of cell size and cell number of spikelet hulls in the transverse direction was performed as described previously. The prepared sections were observed using a microscope imaging system (BX53, Olympus, Tokyo, Japan). Analysis of cell size and cell number of spikelet hulls in the longitudinal direction was done using scanning electron microscope (HITACHI S-3000N, Tokyo, Japan). The cell length in each sample was measured using ImageJ software.

*RNA Extraction and RT-qPCR:* To analyze expression pattern of *OsMADS47,* total RNA was isolated from various tissues of NIP using RNAprep Pure Plant Kit (Tiangen, Beijing, China). First-strand cDNA was synthesized from 1 μg RNA using a reverse transcription kit (R223, Vazyme Biotech, Nanjing, China). RT-quantitative PCR was conducted on ABI7500 Real-time PCR system using SYBR Green qPCR mix (Q331, Vazyme Biotech, Nanjing, China) following the manufacturer’s instructions. The rice *Ubiquitin* gene was used as an internal control to normalize the gene expression data. To analyze the differential expression of genes regulating grain shape and cell cycle, young panicles of 1-2 cm in length were sampled from *OsMADS47* overexpression, *OsMADS47* knockout and NIP plants. For each sample, at least three biological replicates were measured. The RT-qPCR primers used are provided in Supplementary Table 1.

*Flow Cytometric Analysis:* Young panicles of 1-2 cm in length from *OsMADS47* overexpression plants and NIP were sampled and chopped in ice-cold Galbraith’s buffer. The crude extracts were filtered through a 300-mesh Nylon filter to remove the cell debris. The isolated nuclei were stained with propidium iodide (50 μg ml^-1^) and then subjected to flow cytometric analysis (FACSAria II flow cytometer; BD Biosciences, San Jose, CA, USA). In each sample, at least 10,000 nuclei were recorded and analyzed by MODFIT LT software.

*Yeast Two-hybrid Assays:* The full-length CDS of *OsMADS47* was amplified and inserted into the pGADT7 vector and used as the prey. The grain size regulatory genes (*OsMPK3*, *OsMPK6*, *PPKL1*, *PPKL2*, *PPKL3*, *GW8*, *BZR1* and *DLT* etc.) were chosen according to the previous studies and cloned into the pGBKT7 vector using primers listed in Supplementary Table 1. The pGADT7-OsMADS47 and pGBKT7 derivatives were co-transformed into the yeast strain AH109 and cultured on SD/-Trp-Leu medium (Clontech, Mountain View, CA, USA) for 3 d at 30 °C. Then, interaction between bait and prey was examined on the selective medium SD/-Trp-Leu-His-Ade. The combinations of pGADT7-OsMADS47 with the empty pGBKT7 and pGBKT7 derivatives with the empty pGADT7 were used as the negative controls.
